# Implementation of a comprehensive template to support personalised care for people with multiple long-term conditions: a mixed-methods evaluation in primary care

**DOI:** 10.1136/bmjopen-2025-102325

**Published:** 2026-04-15

**Authors:** Rachel Johnson, Andrew Turner, Clare Jinks, Mari Carmen Portillo, Caroline Michaela Coope, Alice Louise Moult, Kate Alice Lippiett, Dereth June Baker, Cindy Mann, Lauren J Scott, Krysia Dziedzic, Zoe Paskins, Richard Byng, Simon Chilcott, Grace Scrimgeour, Chris Salisbury

**Affiliations:** 1 Bristol Medical School, University of Bristol, Bristol, England, UK; 2 NIHR ARC West, Bristol, UK; 3 School of Medicine, Keele University, Keele, UK; 4 Faculty of Health Sciences, University of Southampton, Southampton, UK; 5 NIHR ARC Wessex, Southampton, UK; 6 Keele University, Newcastle-under-Lyme, UK; 7 School of Medicine, Keele University, Newcastle-under-Lyme, UK; 8 Peninsula Medical School, Plymouth University, Plymouth, UK

**Keywords:** Primary Health Care, Multimorbidity, Implementation Science, Delivery of Health Care, Integrated, Patient-Centered Care, QUALITATIVE RESEARCH

## Abstract

**Abstract:**

**Background:**

Healthcare services are mainly organised around single health conditions and need reconfiguration to meet the needs of people with multiple long-term conditions (multimorbidity). Typically, people are offered annual reviews for each of their long-term conditions separately. In a randomised controlled trial, a comprehensive computerised template based on a personalised care model increased the person-centredness of multimorbidity reviews in primary care, but there were implementation challenges. We sought to understand and address the challenges of implementing a template to support personalised primary care for people with multimorbidity (PP4M).

**Objectives:**

To explore the extent of implementation and factors influencing uptake of the PP4M intervention. To understand factors influencing implementation and normalisation of the template.

**Design:**

Convergent parallel mixed methods within a non-randomised hybrid implementation-effectiveness study. Normalisation Process Theory (NPT) informed design, data collection and analysis.

**Setting:**

Primary care (general practices) in three English regions.

**Participants:**

Quantitative: Patients aged 18 years or over and had at least three types of long-term conditions (routine data collection); staff involved in using the template in implementation practices (Normalisation MeAsure Development (NoMAD) questionnaire).

Qualitative: Staff at implementation practices.

**Intervention:**

A multimorbidity computerised template to support personalised annual reviews. NPT-informed implementation package delivered to implementation practices included: process mapping, software support and training.

**Data collection:**

Routine medical record data; NoMAD questionnaires and qualitative interviews in implementation practices.

**Primary/secondary outcomes:**

Measures of reach, fidelity, acceptability and sustainability.

**Analysis:**

Quantitative data: descriptive statistics, logistic regression and difference-in-difference models. Qualitative data analysis conducted using NPT coding manual.

**Results:**

In practices that received an NPT-informed implementation package, use of the template increased more, across patients with a range of demographics and health conditions, than in those that did not receive the implementation package (OR 2.86 (95% CI 2.34 to 3.49)). The implementation package successfully triggered NPT processes of coherence and cognitive participation, and, to a lesser extent, collective action and reflexive monitoring. Contextual factors, including a lack of staff generalist skills and disease-specific incentives, impeded engagement and sustained implementation.

**Conclusions:**

Focusing on the processes of normalisation as mechanisms of implementation facilitated development of an implementation strategy with potential to trigger those mechanisms, but did not sufficiently address contextual factors. Implementation strategies to support personalised care must consider wider system and practice level contextual factors, such as incentives and staff training.

**Trail registration number:**

https://doi.org/10.1186/ISRCTN40295449 (2022–08-03, retrospectively registered.)

**Strengths and limitations of this study**

- This mixed-methods implementation study addresses the implementation challenges identified in the 3D randomised controlled trial of whole person reviews for people with multimorbidity which may have contributed to the lack of impact of the intervention on the trial’s primary outcome.
- General practitioner practice recruitment was focused in areas of higher socio-economic deprivation, where the prevalence of multimorbidity is greatest, allowing us to highlight the challenges faced by these practices in implementing comprehensive multimorbidity care.
- We used implementation theory, specifically Normalisation Process Theory (NPT), within a realist approach to inform the implementation plan, data collection and analysis and report one of the first studies to describe the use of the NPT coding manual.
- Through the use of mixed methods, we developed a more comprehensive understanding of the implementation of the PP4M (personalised primary care for people with multimorbidity) template than would have been obtained through analysis of quantitative and qualitative data separately.
- Because this is a non-randomised study, there could be unmeasured differences between implementation and control practices, such as patient and/or staff characteristics, which we were unable to account for and could have therefore impacted on our conclusions.

## Introduction

 An increasing number of people live with multiple long-term conditions (multimorbidity).^1^ Having multimorbidity is associated with reduced quality of life and increased healthcare costs, and affects people in socio-economically deprived areas at younger ages.^2 3^ People with multimorbidity report that their care is poorly coordinated and insufficiently focused on what matters to them.^4^ Healthcare services are often organised around single conditions, requiring reconfiguration to better meet the needs of people with multimorbidity.^5^

International healthcare organisations^6 7^ emphasise the need for care for long-term conditions to be better organised around what matters to people. Recommended care elements for people with multimorbidity include care coordination, shared decision-making and assessment of social factors.^8^ In the UK, the National Health Service Comprehensive Model for Personalised Care^9^ includes supported self-management, care and support planning, shared decision-making and links to social prescribing. However, it is unclear how best to implement this model in primary care for people with multimorbidity.

In UK general practice (GP), care for some long-term conditions is incentivised by the Quality and Outcomes Framework (QOF), a pay-for-performance framework by which practices are paid for meeting quality targets for specific health conditions.^10^ Typically, individuals with long-term conditions included in the QOF are reviewed annually, each condition reviewed in separate appointments using disease-specific computer templates. Providing comprehensive reviews of all an individual’s long-term conditions together could improve healthcare efficiency, quality of care and patient experience.

Recent systematic reviews of the effectiveness of interventions for the management of multimorbidity in primary care and community settings have concluded that the effectiveness of existing interventions remains uncertain,^11 12^ with no consistent evidence of improved outcomes for different intervention components, and paucity of data on health outcomes and healthcare costs. A previous systematic review of interventions designed to involve older people with multimorbidity in their care identified a large gap between clinical guidelines for multimorbidity and an evidence base for implementation of their recommendations.^13^ To date, few studies focus on the factors affecting implementation of personalised care approaches for multimorbidity in real-world primary care settings.

In the three-dimensional (3D) randomised controlled trial,^14^ a comprehensive computerised template based on a personalised care model was used to support healthcare professionals to review all conditions simultaneously in people with multimorbidity. This approach improved the person-centredness of multimorbidity reviews, but not quality of life. However, difficulties with implementation were identified. These included problems re-organising recall systems, insufficient staff training and challenges in embedding and normalising changes to processes of care within a randomised controlled trial.^15^ Despite the potential for improving person-centredness, the templates and strategies developed for research studies such as the 3D trial have not been widely adopted.

To address these problems, we aimed to evaluate the implementation of a comprehensive template package to support personalised annual reviews for people with multimorbidity in UK primary care, in routine care outside of a randomised trial. We worked with a commercial company (Ardens)^16^ to refine an existing multimorbidity template and developed a theoretically informed implementation strategy.

In this paper, we report a mixed methods evaluation designed to answer the following research questions: (1) To what extent was the personalised primary care for people with multimorbidity (PP4M) intervention implemented and normalised? (2) What factors influenced implementation and normalisation of the PP4M template?

## Methods

### Objectives

#### Quantitative

To explore the extent of implementation and factors influencing uptake of the PP4M intervention.

#### Qualitative

To understand factors influencing implementation and normalisation of the template.

### Design

Convergent parallel mixed-methods within a non-randomised hybrid implementation-effectiveness study. Quantitative and qualitative data collection and analyses proceeded in parallel, before the results were merged to provide greater insight into implementation. The quantitative and qualitative methods have equal priority. This paper evaluates the implementation of the PP4M template from the perspective of GP and is reported using STARi (Standards for Reporting Implementation Studies)^17^ and GRAMMS^18^ (Good Reporting of A Mixed Methods Study) checklists (see online supplemental additional file 1).

### Setting

GPs in three geographical areas in England (Bristol, Southampton and Staffordshire). Practices in deprived areas were prioritised, because multimorbidity is more prevalent in these areas.^3^ Template implementation occurred in 2022–2023.^19^

### Theoretical and conceptual frameworks

Normalisation Process Theory (NPT) seeks to understand how innovations become normalised in healthcare settings.^20^ ^21^ We chose NPT because it is an effective and flexible method for understanding implementation of interventions in complex and resource-constrained healthcare settings such as UK primary care, particularly those focused on the management of long-term health conditions and multimorbidity.^22^ We used NPT to inform the development of the implementation strategy, data collection and analysis. Quantitative data collection included the Normalisation MeAsure Development (NoMAD) questionnaire,^23^ a 23-item survey for assessing implementation of complex interventions in healthcare from the perspective of professionals directly involved.

**Figure 1:**
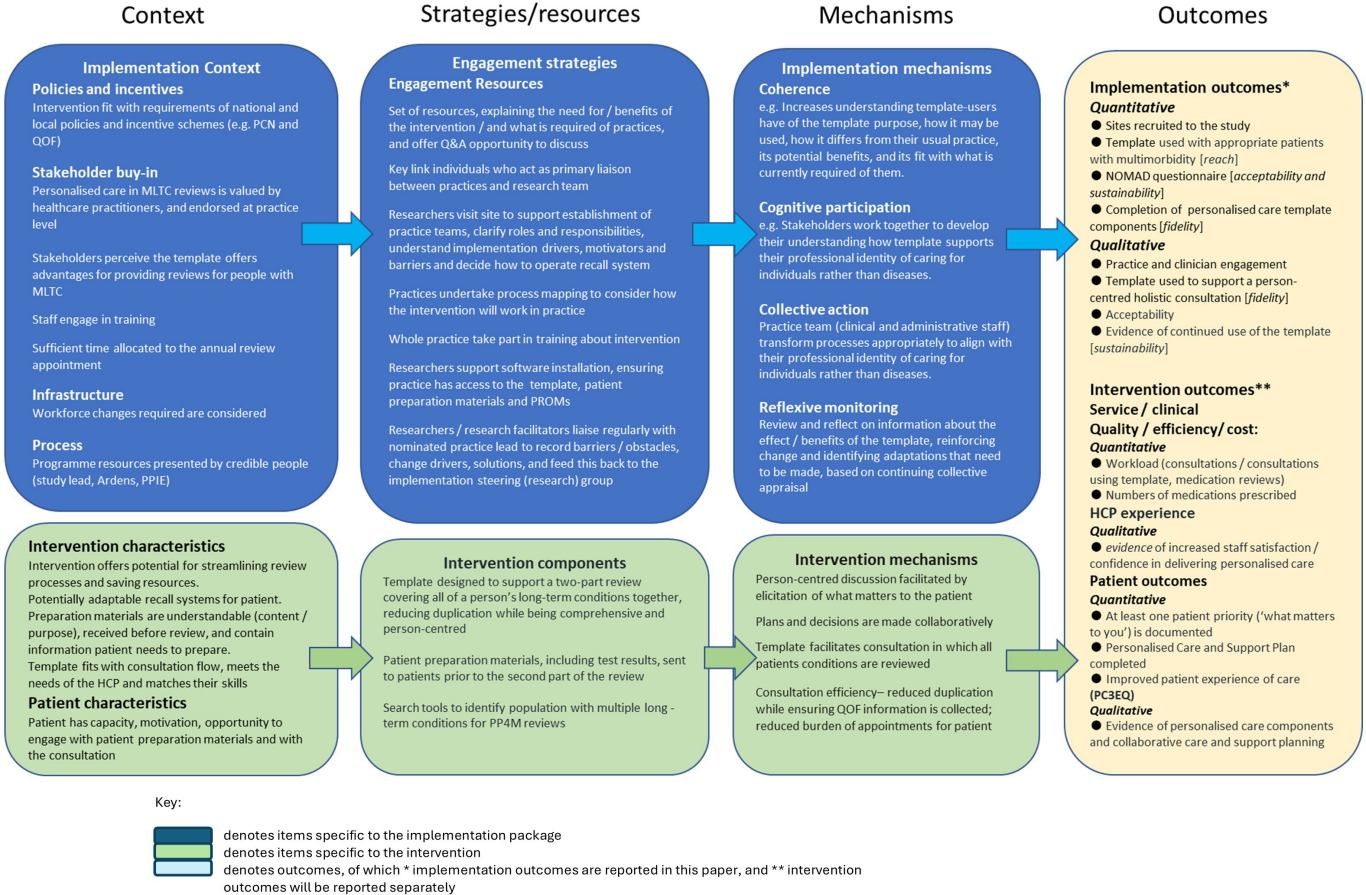
Logic model. HCP, healthcare professional; MLTC, Multiple Long Term Condition; NoMAD, Normalisation MeAsure Development; PCN, Primary Care Network; PP4M, personalised primary care for people with multimorbidity; PPIE, Patient and Public Involvement and Engagement; PROMs, Patient Reported Outcome Measures; PC3EQ, Person-Centred Coordinated Care Experience Questionnaire; Q&A, question and answer; QOF, Quality and Outcomes Framework.

NPT also guided the development of qualitative interview topic guides. For qualitative analysis, we used May *et al*’s NPT coding manual which combines NPT with the Context-Mechanism-Outcome (CMO) configuration of realist evaluation studies.^24^ ^25^ The manual conceptualises the four original NPT constructs (coherence, cognitive participation, collective action and reflexive monitoring) as the mechanisms by which normalisation is achieved and adds further constructs relating to normalisation processes within contextual and outcome domains. In line with the NPT coding manual, our initial logic model (figure 1) described the contextual factors we considered when developing the implementation strategy and intervention, the mechanisms by which both were anticipated to act and the outcomes by which each would be evaluated.

### Intervention

The PP4M intervention comprises a computerised template and individualised patient preparation materials and was available to both implementation and control practices. Only implementation practices received the ‘implementation package’ described below.

The PP4M template was developed from an existing template to provide improved functionality for the assessment of multiple conditions, with new components added to support comprehensive, holistic assessment and promote personalised care. The assessment included questions about what matters most to the patient, quality of life, pain, function and mental health. The interactive template only prompts actions or displays information relevant to the individual’s conditions. The intention is that the template is delivered over two extended consultations, instead of multiple, shorter consultations for individual conditions. During the initial consultation (usually conducted by a healthcare assistant or nurse), the template supports the collection of information and assessments relevant to the person’s long-term conditions, including arranging necessary tests and meeting QOF requirements. The annual review (usually conducted by a nurse or GP) should involve discussion of the results from the initial consultation, a medication review (if required) and agreeing a care and support plan with the patient. Patient preparation materials, developed by the Year of Care partnership,^26^ are written documents tailored to individual patients, presenting and explaining the results of relevant recent tests and prompting people to consider what they want to discuss at their annual review appointment. Three implementation practices in one area received extra training in personalised care from the Year of Care partnership.

#### Implementation package

**Figure 2:**
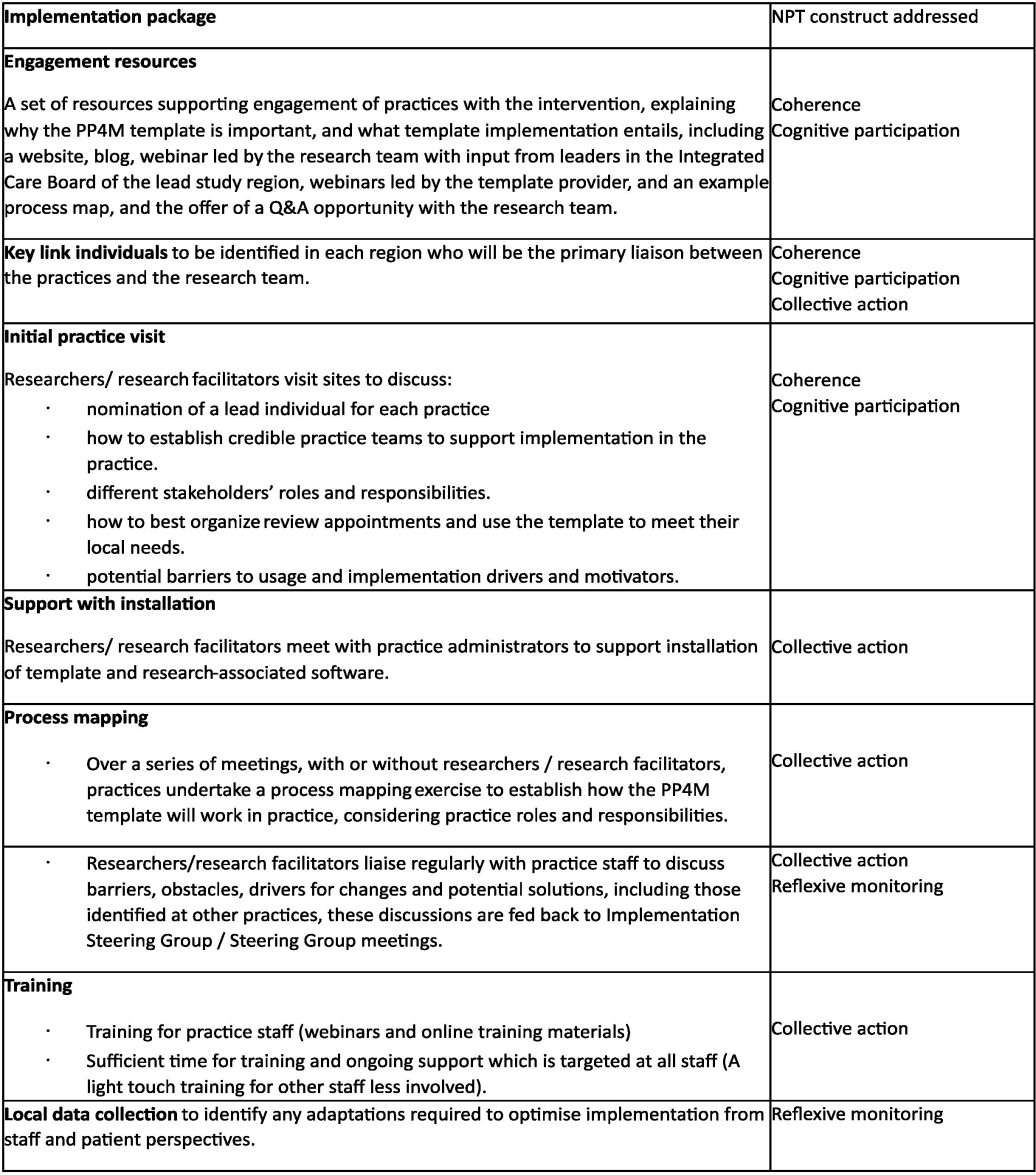
Implementation package mapped to NPT constructs. NPT, Normalisation Process Theory; PP4M, personalised primary care for people with multimorbidity; Q&A, question and answer.

The implementation package was informed by NPT, a Knowledge Mobilisation Toolkit for Primary Care,^27^ and the research team’s experience of researching and delivering implementation in primary care, including in the 3D study.^15 28^ The implementation package included resources explaining the intervention purpose and requirement of practices, researcher-supported process mapping, software support, training and ongoing implementation support. In figure 1, the logic model illustrates how the implementation strategy was proposed to influence implementation outcomes, while figure 2 maps implementation package components to the four main NPT constructs.

### Practice sampling

Practices were eligible if they used EMIS electronic patient record systems,^29^ had ≥5000 registered patients and access to Ardens templates (available here: https://support-ew.ardens.org.uk/support/solutions/articles/31000161582-multi-morbidity-templates). 156 practices meeting our eligibility criteria were invited to express interest in taking part as implementation practices. 38 practices responded. After initial discussions with these practices about what the study involved, we selected 16 ‘implementation’ practices to represent the three study regions and to ensure at least half the practices were from areas of above-average deprivation. Following recruitment of implementation practices, 16 eligible practices in the same regions were recruited as controls, seeking balance in region, deprivation and list size.

No formal sample size calculation was made. Intervention and control practices were recruited with the intention of obtaining wide variation across practices in practice size, urban/rural location and deprivation and ethnicity of patient populations.

### Quantitative data collection and analysis

#### Routinely collected healthcare records data collection and analysis

Patients aged 18 years or over with at least three types of long-term conditions were eligible for quantitative analysis (see online supplemental additional file 2 for list of conditions). Implementation practices chose which people with multimorbidity to prioritise for annual reviews using the template.

Routinely collected healthcare records data (collected as part of standard medical care) were extracted in July 2023 for two time periods: pre-implementation period (1 January 2021 to 31 March 2022) and implementation period (1 April 2022 to 30 June 2023). To compare implementation and control practices, we included patients who were registered at the practice at all three time points (1 January 2021, 1 April 2022 and 30 June 2023).

Data analysis is summarised below and described in detail in online supplemental additional file 3. We considered two populations for analysis. First, all eligible patients, regardless of template use, were considered, similar to an intention-to-treat analysis. This is the analysis described unless otherwise specified. Second, we considered patients from implementation practices in whom the initial and/or main template had been used in the implementation period, and patients from control practices where neither the initial nor annual review templates had been used with any eligible patients in either time period; (hereafter referred to as a per-protocol analysis).

### Demographic data and long-term conditions

Age, sex, Index of Multiple Deprivation (IMD) decile,^30^ ethnicity and number and type of long-term conditions were extracted at patient level for all eligible patients.

### Assessment of implementation outcomes

Lists of clinical codes (recorded as part of GP consultations) corresponding to key template processes were extracted anonymously from the medical record.

#### Reach

We extracted codes for use of the PP4M template (initial consultation, annual review or both), in implementation and control practices in the implementation period. The effect of patient characteristics on use of the template (initial consultation or annual review) was assessed in the implementation period in implementation sites at the patient level using multilevel logistic regression with practice as a random effect, and adjusted for demographics (age, sex, ethnicity and IMD) as fixed effects. Further models added each long-term condition in turn, to assess differences in template use by condition.

#### Fidelity

The proportion of eligible participants in whom key personalised care template elements were coded in the pre-implementation and/or implementation period are presented descriptively. Using multilevel logistic regression, we assessed whether use of personalised care sections of the template differed between pre and implementation periods, adjusting for control practices and between intervention and control practices, in a difference-in-difference framework. Models were adjusted for demographics (as above) as fixed effects, and patient ID nested with practice ID as random effects. Fidelity was further modelled at the practice level as the percentage of patients at each practice with the outcome.

#### Acceptability and sustainability

All staff involved in using the template in implementation practices were invited to complete the NoMAD questionnaire 3–6 months after their practice started using the template. Acceptability was assessed in relation to the four main NPT constructs. Sustainability was assessed using three specific questionnaire items. These are reported descriptively by staff group and by use of the template (from never used to used >15 times).

Data management and analyses were performed in Stata V.17.0.

### Qualitative data collection and analysis

Staff at implementation practices were purposively sampled to capture different ways practices implemented the template, and different staff roles. Potential participants were sent an invite by the research lead at the practice, on behalf of the study team. Interviews took place between October 2022 and October 2023. All participants provided either written or verbal (recorded) informed consent. Interview topic guides were informed by the NPT constructs of coherence, cognitive participation, collective action and reflexive monitoring plus components of personalised care. The topic guide explored: (1) how the new review process operated within the practice, including workflow changes, benefits, challenges and impacts; and (2) for those directly conducting reviews, how they used the review template, its usability and its perceived influence on the conduct and quality of patient reviews. Researchers kept detailed written field notes of meetings with implementation practice. Interviews were transcribed verbatim and anonymised. Data were managed in NVivo V.1.6.1.

The 12 primary constructs of the NPT coding manual were used for analysis.^25^ Researchers familiarised themselves with transcripts and the NPT constructs and met regularly to ensure consistent use of the codes, and to discuss interpretation of the data. Data were coded deductively to the 12 NPT constructs, with additional inductively developed codes (abductive coding). Data for each code were summarised into a set of summary propositions (statements describing the developing findings), with accompanying quotes. Field notes were read to identify evidence to support propositions or suggest new propositions. Propositions were grouped, and themes developed, and through team discussion, including with the public collaborators, the key messages were developed. Themes developed from the data coded to the four constructs relating to ‘Context’ and the four constructs related to ‘Outcomes’ are presented in the ‘Context’ and ‘Outcomes’ sections of the results, respectively. An example of qualitative data coding is given in online supplemental additional file 4.

### Mixed methods analysis

Qualitative and quantitative analysis proceeded in parallel. The combined results and the implications of the analysis were considered by the multidisciplinary research team, including the public collaborator. Findings were discussed in relation to the logic model, and the implications for the contextual factors, mechanisms and outcomes of the logic model are presented. Quantitative and qualitative results were considered side-by-side; the extent to which the data converged/diverged was discussed with analytic insights developed through discussion.^31^

### Patient and public involvement

Our public co-applicant SC, who has lived experience of multimorbidity, contributed to study design and oversight as a member of the co-investigator group. Public contribution was led by an academic expert in public involvement (CM). Public collaborator groups in each study region met regularly for within-region and between-region groups throughout the study, providing feedback on the project design and evaluation measures and helping to develop and pilot participant materials and interview topic guides. Emerging results were discussed at several points with public contributors who helped to shape key messages for dissemination. Further details of their involvement have been published elsewhere.^32 33^

## Results

**Figure 3:**
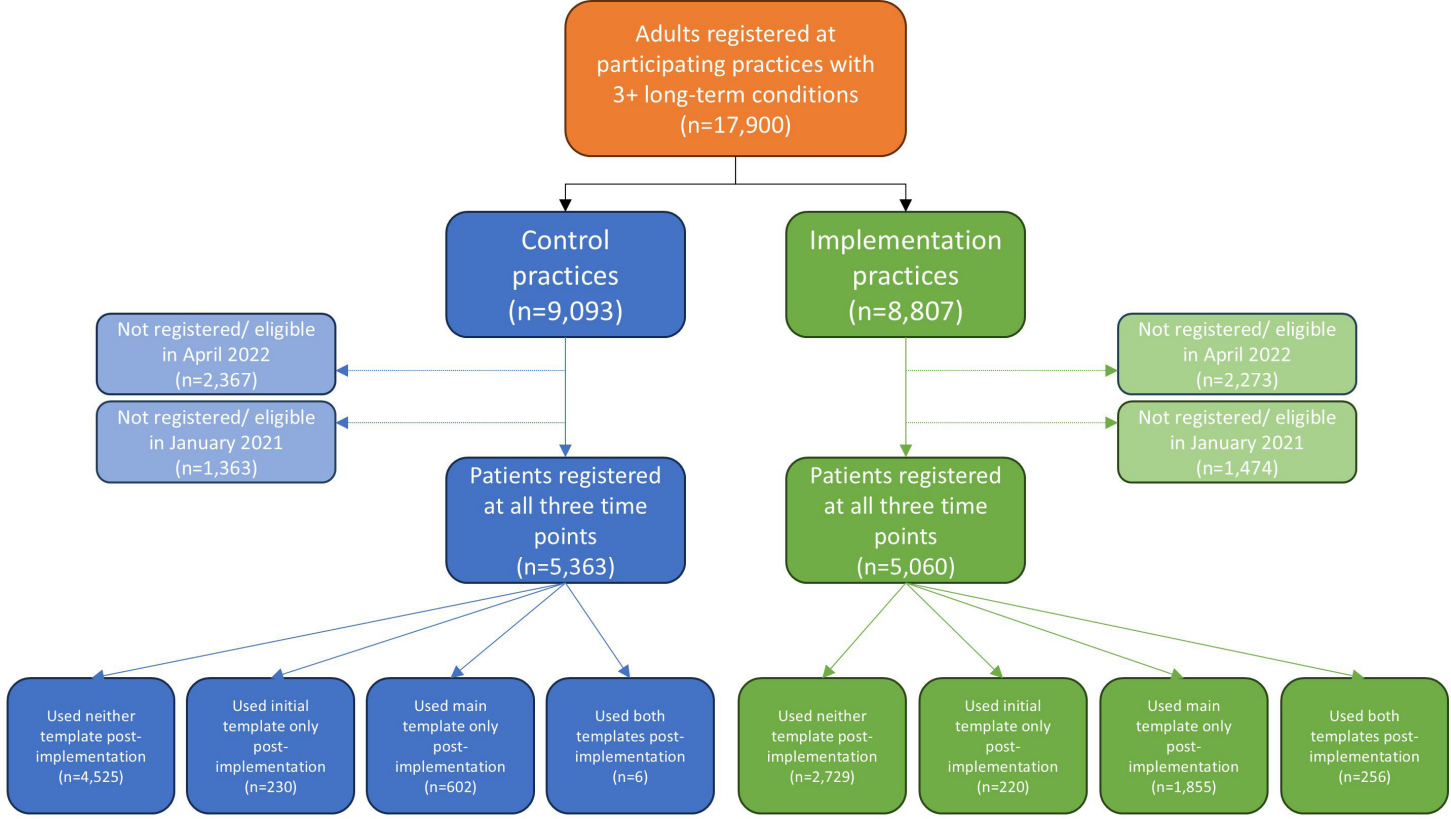
Flow of participants at implementation and control practices.

Figure 3 shows the flow of patients at the 16 implementation and 16 control practices.

Practice characteristics are described in table 1. All implementation and control practices contributed quantitative data. 14 implementation practices contributed qualitative data.

**Table 1:** Practice characteristics

	Implementation practices (n=16)	Control practices (n=16)	National average (England)
Practice characteristics			
List size (mean) (median, IQR)*	15 461 (11 237–7030)	16 409 (14 980–9153)	10 001
Number of healthcare assistants per practice (mean, SD)†	2.9 (2.11)	2.7 (1.74)	1.5
Number of practice nurses or nurse practitioners per practice (mean, SD)†	8.6 (5.74)	8.1 (3.77)	3.7
Number of fully qualified GPs per practice (mean, SD)†	9.6 (8.28)	10.7 (6.46)	5.9

* Data from: https://digital.nhs.uk/data-and-information/publications/statistical/patients-registered-at-a-gp-practice/april-2024.

† Data from: https://digital.nhs.uk/data-and-information/publications/statistical/general-and-personal-medical-services/31-may-2023.

GPs, general practitioners.

Table 2 summarises qualitative participants. Quantitative and qualitative findings are presented sequentially under the headings of implementation context, mechanisms and outcomes.

**Table 2:** Summary of qualitative participants (number per practice, professional group)

Region	List size*	IMD quintile	Professional group						Total per practice
			Management	Administrative	Healthcare assistant	Nursing	GP	Social prescriber	
1	M	5	2		1				3
1	S	5				1		1	2
1	M	3		1	2	3	1		7
1	S	4	1		1	1			3
1	M	5	1			1			2
1	S	1	1			1	1		3
1	L	4			1	4			5
2	M	1		1	1	1			3
2	M	4		3		3	1		7
2	L	4	1	1		2	1		5
3	M	3	1				1		2
3	S	4		1		2			3
3	M	2				1	1		2
3	M	2		1	1				2
Total across practices			7	8	7	20	6	1	49

* S/M/L: (Small <10 000; Medium 10 000–20 000; Large>20 000).

GP, general practitioner; IMD, Index of Multiple Deprivation.

### Implementation context

#### Quantitative

##### Practice characteristics

Participating GPs were slightly larger with more staff compared with average English practices (table 1).^34 35^

##### Patient characteristics

Patient characteristics are reported in online supplemental additional files 5 and 6. In short, 20.4% patients were aged 18–59 year and 79.6% were 60+ years, 55.3% were female, 88.1% were of white ethnicity and 52.9% were in IMD quintiles 1–2 (least deprived); we obtained complete age and sex data for all patients. However, <0.1% (seven participants) were missing IMD and 6.2% were missing ethnicity. Multimorbidity was common: 4.6% of adult patients in implementation practices had multimorbidity (defined as at least three types of long-term conditions, see online supplemental additional file 2 for list of conditions), rising to 20.8% in patients aged 80+ years. Almost all had at least one cardiovascular diagnosis, and over half had diabetes, respiratory disease and/or a mental health diagnosis. More than half of the patients in implementation practices came from areas of above-average deprivation.

### Qualitative

**Figure 4:**
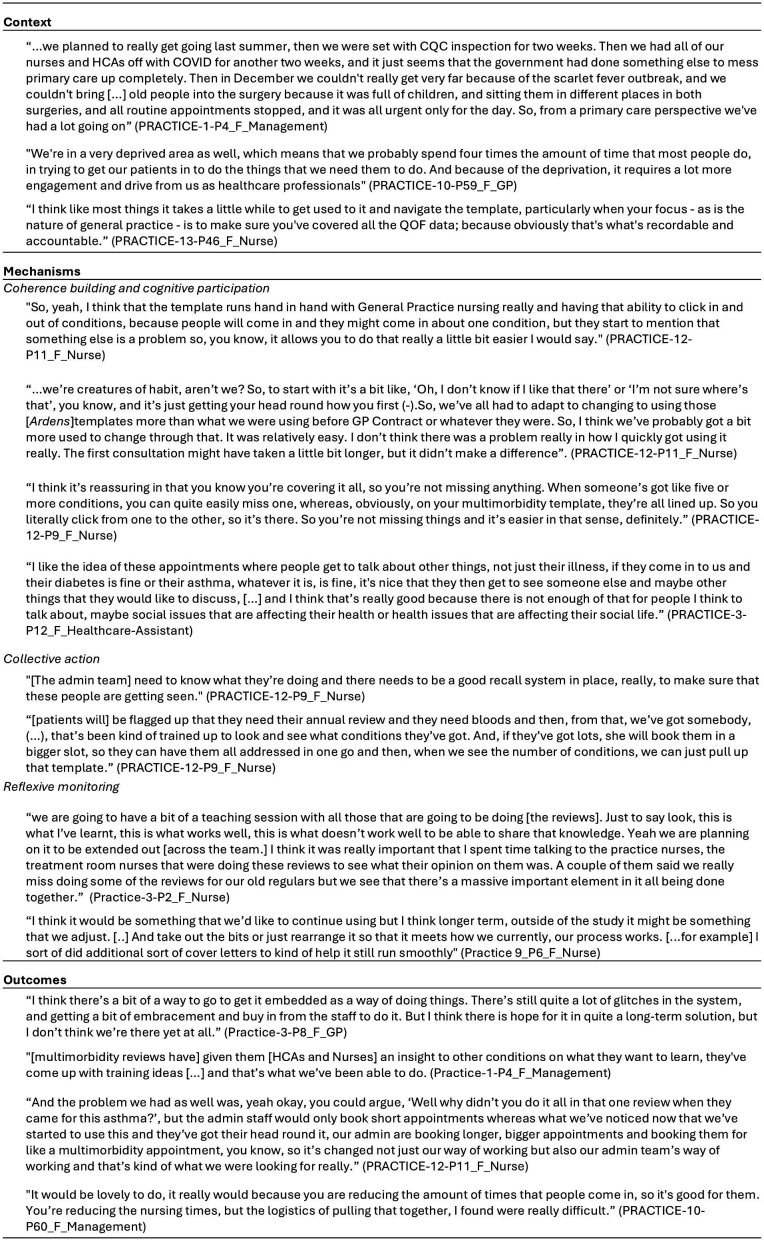
Illustrative quotes: context, mechanisms, outcomes. CQC, Care Quality Commission; GP, general practitioner; HCAs, healthcare assistants; QOF, Quality and Outcomes Framework.

(see figure 4 for illustrative quotes.)

#### Motivations for implementation

The intervention was perceived to address two important challenges. First, practices recognised the template’s potential to help meet QOF requirements, by standardising the recording of remunerated care processes. The ability of the template to support meeting QOF requirements was frequently discussed as essential.

Second, practice staff were inherently motivated to make the experience of patients with multimorbidity more personalised and holistic and recognised the template’s potential to support this. Staff perceived that existing care could be inefficient for people with multimorbidity and for staff, requiring multiple appointments with different practitioners for repetitive processes. Anticipated benefits of the template included fewer, more efficient appointments and improved interactions with patient populations not currently well engaged with healthcare providers.

#### Challenges to implementation

Implementation practices faced wider system challenges affecting their ability to transform existing administrative and clinical processes to implement the template. Challenges included ongoing changes to practice systems since the COVID-19 pandemic, practice mergers, staff shortages and excessive demand. Practices in deprived areas were particularly impacted by these challenges and served populations with complex needs who required additional efforts to support their engagement with healthcare.

#### Skills of the primary care team

Using the multimorbidity templates across a range of medical conditions required a breadth of skills and capabilities. The numbers of healthcare assistant/nursing staff available, with the appropriate level of training or range of skills required, varied across practices and crucially impacted their ability to implement the new system. Often, nurses had developed advanced skills in the management of specific long-term conditions. Lack of confidence in dealing with other long-term conditions, or reluctance to do so without advanced skills, made multimorbidity care difficult. The advantage of having a nursing workforce with generalist skills was recognised to provide flexibility in how care could be provided.

#### Fit with existing IT and administrative processes

The extent of administrative and information technology (IT) changes required to implement the new system depended on existing practice systems for organising long-term conditions reviews, and the skills of staff in implementing IT system changes. Practices varied in the extent to which they were able to administer longer multimorbidity appointments flexibly, despite potential long-term efficiencies. The general layout and functionality of Ardens templates was familiar to practice staff, facilitating implementation.

#### Practice change processes

The extent to which wider practice teams worked together to plan implementation of the new system for annual reviews varied. Having engaged practice staff who supported use of the intervention, protected time for supporting change processes and involving the wider staff team in planning changes increased buy-in and supported implementation. Process mapping provided an opportunity for practice staff to consider the fit of the system within their current processes, and any changes, including to IT and administrative systems, and staff roles, that needed to be made to support implementation.

### Implementation mechanisms

We proposed the four main constructs of NPT (coherence, cognitive participation, collective action and reflexive monitoring) as the mechanisms by which the implementation package would support implementation of the PP4M template (figure 1). Here we report quantitative (NoMAD questionnaire) and qualitative findings relating to the four main NPT constructs.

#### Quantitative: NoMAD questionnaire

56 staff from 13 practices completed the NoMAD questionnaire. Agreement with statements about coherence was generally high, particularly among nurses and healthcare assistants (online supplemental additional file 7). There was stronger agreement for cognitive participation for all staff groups. There was overall agreement on collective action, but less strong agreement in specific questions about the sufficiency of training and resources to support use of the template. There was positive agreement on reflexive monitoring from healthcare assistants and to a lesser extent from nurses, but more neutral opinions were expressed by other staff. Staff who had made greater use of the template were more positive about reflexive monitoring than other staff (online supplemental additional file 8). Other NoMAD domains were not strongly related to use.

#### Qualitative findings

(see figure 4 for illustrative quotes).

##### Coherence building and cognitive participation

These two NPT constructs are reported together because they often overlapped in respondents’ interviews. Staff built knowledge and understanding of the template through the implementation package resources, interactions with colleagues and through trying out the template in training sessions or on their own, with dummy patients. Through these, staff tested and internalised their understanding of the rationale for the template, and how it could benefit patients and improve practice efficiency. Process mapping provided an important opportunity for practice staff to develop their understanding. Clinicians exhibited varied attitudes and approaches to using the template, influenced by their clinical skills, experience and confidence, familiarity with other templates and personal practice style.

Staff appreciated that the template was comprehensive, simple to navigate, collated required information in one place, only showed pages relevant to the patient’s health conditions, reduced duplication, included space for recording what was important to the patient, included medication review, produced a well-structured report in the clinical record and improved the consistency of coding of key processes. Some nurses appreciated that, if a patient had attended for an initial consultation, their subsequent appointment with a nurse could be unencumbered by tests and assessments. Some practitioners also identified limitations of the template, for example, gains in streamlining may come at the cost of lack of detail compared with the single disease templates they were familiar with.

For some nurses, shifting their focus from disease management to overall well-being support presented challenges, including professional anxiety and difficulty in shifting from task-oriented to person-centred care. There was a range of views on the template’s depth, complexity and how it supported the healthcare professionals’ ability to promote person-centred care, leading individuals to prioritise sections selectively. Absence or selective implementation of some key parts, like patient preparation documents, indicated challenges in achieving comprehensive use or perceiving benefits as sufficient.

##### Collective action

Effective administrative processes were critical for the template’s integration and for managing multimorbidity reviews, for example, booking and coordinating initial and main review appointments; ensuring appointments were of appropriate length and with a practitioner with the right skills to review multiple conditions. Some practices adopted appointment booking systems that required individual tailoring of processes rather than automation, making it difficult to normalise the intervention for larger numbers of patients.

Practices trialled different ways of providing multimorbidity reviews, using different combinations of one or two appointments, seeing different staff, and with/without patient preparation documents. Some practices instituted dedicated clinics for multimorbidity reviews, and in one practice, a ‘one stop clinic’ was introduced where the patient saw a series of different nurses, each covering different parts of the review. However, they found this took longer and, as a result, these clinics were stopped.

##### Reflexive monitoring

Some reflected on perceived benefits of the template for patients (eg, encouragement of person-centred care, fewer visits to the practice) and for staff (eg, improved job satisfaction resulting from longer patient appointments), and sections of the template that worked less well. The template was perceived to be more comprehensive for managing certain long-term conditions (eg, asthma) than others (eg, mental health, learning disabilities). Some practices were able to make changes to their processes to better fit the skills of their nursing team and perceived needs of patients. For example, in one practice, GPs were brought into the review process because of an identified need for prescribing capabilities within the review.

### Implementation outcomes

#### Quantitative

##### Reach

There was high usage of the template (initial and/or annual) across implementation practices in the implementation period; the template was used with almost half (46.1%) of eligible patients (online supplemental additional file 9). This was a marked increase compared with pre-implementation (online supplemental additional file 10a). Comparing pre-implementation and implementation periods, there was a much greater increase in template use in implementation practices than in control practices (OR 2.86 (95% CI 2.34 to 3.49), p<0.001, mean difference in percentage 19.45 (7.20 to 31.69)).

The patients in whom the template was used were broadly similar to the cohort of all eligible patients (online supplemental additional file 6). The template was used with patients across the range of sociodemographic characteristics, and across all conditions assessed. It was less well used in younger patients, those in least deprived areas and in eligible patients with epilepsy, mental health problems, dementia or learning difficulties, but more likely to be used in patients with cardiovascular disease (CVD) or with diabetes (online supplemental additional file 9).

##### Fidelity

The implementation practices mostly only used the annual review template, not the initial consultation template. Overall, relatively few of the eligible patients (5.1%) in implementation practices had the two-part review intended (online supplemental additional file 9).

We intended that the template would prompt clinicians to include elements of personalised care not necessarily covered in usual QOF-focused reviews. However, there was low use of some of these elements, and very low usage of questions about mood and relating to care planning (online supplemental additional files 10a and 10b).

Data collection of several items facilitated by the template improved in all practices, but improved more in implementation practices, from a much lower base. Implementation practices had a greater improvement in assessment of mobility, memory, falls and pain (table 3). Several items improved similarly in both implementation and control practices, including frailty assessment, medication adherence and social prescribing.

**Table 3:** Comparisons between implementation and control practices in use of the template

	OR (95% CI)*	P value	Mean difference in percentage (95% CI)*	P value
All eligible patients (intention-to-treat analyses):†				
Initial and/or main template use	2.86 (2.34 to 3.49)	<0.001	19.45 (7.20 to 31.69)	0.002
Mobility assessed	4.98 (3.76 to 6.61)	<0.001	9.32 (5.94 to 12.70)	<0.001
Activities of daily life assessed	1.97 (0.75 to 5.18)	0.172	2.81 (1.37 to 4.25)	<0.001
Mood assessed	1.21 (0.84 to 1.73)	0.311	0.98 (–0.66 to 2.62)	0.240
Memory assessed	2.79 (2.03 to 3.84)	<0.001	2.02 (0.003 to 4.03)	0.050
Falls assessed	2.02 (1.65 to 2.48)	<0.001	6.42 (2.83 to 10.01	<0.001
Frailty assessed	0.65 (0.54 to 0.77)	<0.001	1.39 (–4.53 to 7.30)	0.646
Pain assessed	3.05 (2.20 to 4.23)	<0.001	5.89 (2.23 to 9.55)	0.002
Medication adherence raised	1.14 (0.96 to 1.35)	0.125	3.61 (–4.91 to 12.13)	0.407
Medication reviewed	0.72 (0.62 to 0.84)	<0.001	–4.64 (–11.24 to 1.97)	0.169
Referred for social prescribing	0.75 (0.61 to 0.93)	0.007	1.69 (–2.45 to 5.82)	0.425
At least one patient goal identified	1.35 (0.77 to 2.38)	0.300	–0.46 (–1.82 to 0.90)	0.505
Per-protocol analyses:‡				
Initial and/or main template use	Not possible as groups chosen based on presence or absence of template use			
Mobility assessed	8.92 (6.14 to 2.96)	<0.001	16.67 (10.07 to 23.28)	<0.001
Activities of daily life assessed	Numbers too small in control group			
Mood assessed	1.57 (0.98 to 2.51)	0.058	0.26 (–3.62 to 4.15)	0.895
Memory assessed	1.53 (0.82 to 2.86)	0.181	8.19 (3.05 to 13.33)	0.002
Falls assessed	3.80 (2.85 to 5.06)	<0.001	13.47 (6.98 to 19.95)	<0.001
Frailty assessed	0.54 (0.42 to 0.70)	<0.001	–7.03 (–23.48 to 9.42)	0.402
Pain assessed	11.18 (6.83 to 18.28)	<0.001	13.45 (6.95 to 19.95)	<0.001
Medication adherence raised	1.68 (1.32 to 2.13)	<0.001	12.85 (–0.45 to 26.15)	0.058
Medication reviewed	0.63 (0.49 to 0.81)	<0.001	–6.96 (–15.95 to 2.04)	0.130
Referred for social prescribing	0.98 (0.72 to 1.33)	0.891	2.65 (–4.32 to 9.61)	0.457
At least one patient goal identified	8.63 (3.60 to 20.69)	<0.001	0.82 (–0.44 to 2.08)	0.200

* The presented ORs and mean differences are the interaction term between time period and intervention/control group from the difference in difference model. That is, the additional effect of the intervention in the post-intervention period, having adjusted for the effect of time period and intervention/control group. Further details of the model specification are provided in online supplemental additional file 3. Full details of the percentages of template use for each variable in each time period and in intervention/control group are shown in online supplemental additional files 10a and 10b.

† n=5060 in implementation practices and n=5363 in control practices.

‡ n=2331 in implementation practices and n=2326 in control practices. Per-protocol patients are eligible patients from implementation practices in whom the initial and/or main template had been used in the implementation period, and patients from control practices where no eligible patients had ever used the initial or main templates in either time period (8 control practices were included in the per-protocol analyses).

##### Acceptability and sustainability: NoMAD

Nurses and healthcare assistants found the template to be familiar and felt it would become a part of their normal work. Other staff were more ambivalent (mainly GPs and pharmacists). There was a clear trend of greater familiarity with greater use of the template. (See online supplemental additional files 7 and 8)

##### Qualitative

With ongoing use, staff recognised that the template process became easier, and they saw clearer benefits. Some staff perceived the template as time-consuming, while others valued the opportunity to have longer appointments which increased job satisfaction. Staff reflected that the template encouraged focusing on what is important to the patient, and that patients liked the new process (reduced visits to the practice).

Ways of working continued to evolve with template use. In some practices, it had become normalised; in others, there was an intention to continue to use it but to modify the process, while other practices had achieved only limited changes and use of the template. Some practices allocated additional time for PP4M reviews during the study but felt this was not sustainable.

Practices felt patients needed to adjust their expectations of which staff they would see (eg, nurses rather than doctors), and staff would need time to learn new ways of working. This included changing roles and relationships between members of the practice team and wider healthcare team, for example, increased use of social prescribers and mental health workers. Implementing the new template made individual staff and practices review their training needs and how to meet them. In some practices, administrative and nursing staff had developed and expanded their role as part of a team supporting multimorbidity reviews, but in other cases, staff were unwilling to change/expand their roles. (See figure 4 for illustrative quotes.)

## Discussion

In this study, we evaluated the implementation of a comprehensive template to support personalised care for people with multimorbidity in 16 primary care practices in England, over half of which were in areas of above average deprivation. Almost 1 in 20 of the adult population in recruited practices had multimorbidity. Most primary care staff interviewed recognised the need for personalised care for people with multimorbidity. The template was thought to have the potential to facilitate improved, more efficient consultations and to support QOF processes. However, practices faced many contextual challenges which impeded implementation. In practices that received an NPT-informed implementation package, use of the template increased more, across a broad range of patient demographics and long-term conditions, than in practices that did not receive the implementation package. Use of the template as intended, that is, to support a review delivered over two consultations, was uncommon. Use of some holistic care processes (eg, falls assessment) increased with template use, but some personalised care template sections, particularly care planning, were less well used.

Using mixed methods application of implementation theory, including the newly developed NPT coding manual, informed both intervention development and analysis and allowed us to gain a comprehensive understanding of the implementation of the PP4M template from a range of perspectives. The NPT coding manual aims to ‘clarify and simplify NPT for the user and to make it more easily integrated and workable in research’.^25^ We used the coding manual to explain findings across the CMO heuristic. Our team-based collaborative analysis was used to discuss and seek agreement on the interpretation of coding manual domains and subdomains; however, we still found considerable overlap between subdomains. We did not conduct formal sample size calculations in this study and acknowledge that our sample of 56 staff completing the NoMAD questionnaire may impact the reliability of the survey results.

By recruiting half of our implementation practices in areas of higher socio-economic deprivation, we were able to highlight the challenges faced by these practices in implementing comprehensive multimorbidity care. Implementation and control practices were well matched. As practices were not randomly assigned, there is the potential for unrecognised differences between implementation and control practices.

A systematic review of personalised care and support planning for people with single long-term conditions concluded that it has potential to improve some health outcomes, although more studies are needed to understand which groups might benefit.^36^ A prospective cohort study of the implementation of care plans for long-term conditions in UK primary care concluded that the use of care plans in routine care is uncommon, and more proactive attempts at implementation may be required to test their potential.^37^ In the 3D trial,^14^ over 1500 patients from 33 GPs were randomly allocated to a personalised whole person review or usual care. The 3D intervention led to improvements in personalised care. However, many practices struggled to fully implement the new approach. A process evaluation concluded that implementation failure contributed to the lack of effect of the 3D intervention on quality of life (the trial’s primary outcome). Reasons for incomplete implementation included unexpected pressure on practice resources (including staff), implementation choices made by practices (eg, not involving the administrative team) and insufficient training for using patient-centred approaches. Implementation was also affected by the research trial context in which implementation was short-term and applied to only a small proportion of patients in each practice, while others received usual care, increasing the risk of confusion and duplication.

In this study, the four core constructs of NPT were confirmed as mechanisms supporting implementation. In our implementation strategy, process mapping and ongoing interactions with researchers/research facilitators supported these mechanisms. By providing the opportunity to engage in discussion about existing practice processes and the fit of the intervention with practice processes, these implementation strategy components helped practice staff to understand, individually and collectively, how the template worked and how it could be used in practice within an individual or team’s existing work practices, that is, supporting coherence and cognitive participation. However, the extent of wider system challenges such as excessive workload, the challenge of matching the skills of practice staff to the need to provide personalised care for patients with multiple conditions, and reconfiguring existing practice processes to deliver the reviews was not addressed sufficiently by the implementation package, and arguably could not have been addressed at practice level. Contextual factors impacted the extent to which practices, particularly those in areas of high deprivation, had capacity to engage with the implementation strategy, and to make longer-term changes required to sustain implementation. In this study, implementation out with a randomised trial allowed practices to make incremental changes to processes over time. However, the work of taking part in the study remained burdensome for some practices.

We found that the template was more likely to be used in patients with CVD or diabetes, and less likely to be used in patients with epilepsy, mental health problems, dementia or learning difficulties. It is likely that this is because CVD and diabetes are common conditions in which nurses develop advanced skills, whereas fewer primary care practices have nurses with advanced skills in epilepsy, mental health, dementia or learning difficulties. In addition, practices may have existing mechanisms for reviewing patients with some conditions, for example, annual reviews for people with learning difficulties are incentivised by the QOF. The initial consultation template was much less used than the annual review template. This may reflect a lack of resources to deliver, or perceived inefficiency of, two separate appointments.

There is an urgent need to understand how best to deliver personalised care for people with multimorbidity. GP teams recognise the need to improve patient care, but face wider system challenges, lack of capacity to engage in improvement processes and a mismatch between the skills required for multimorbidity care and those existing within the primary care team. Proactive care of long-term conditions in the UK is primarily provided by nurses and healthcare assistants. Healthcare staff consulting with people with multimorbidity need to have skills and confidence to assess and advise people with all conditions, which are common in people with multimorbidity (eg, cardiovascular conditions, respiratory conditions, diabetes and mental health problems) in addition to skills associated with person-centred care, and to undertake training if necessary. Addressing these barriers may take considerable time and resource and probably requires action at a policy and educational level in terms of incentives, contractual requirements and professional training programmes, rather than being remedial at a practice level.

Use of a multimorbidity template is likely to be necessary to provide a more effective and efficient model of care, provided it is implemented with sufficient attention to staff training in person-centred care delivery, practice workload and appropriate incentives. This study has shed light on the challenges of engaging staff in personalised care and support planning. To address this, stronger evidence is needed about the benefits (or otherwise) of care planning and how it can be best delivered.

## Supplementary material

10.1136/bmjopen-2025-102325 online supplemental file 1

## Data Availability

Data are available upon reasonable request.
