# Supplementary material for: Implementation of a comprehensive template to support personalised care for people with multiple long-term conditions: a mixed-methods evaluation in primary care
Source: BMJ Open. 2026 Apr 15;16(4):e102325. doi: 10.1136/bmjopen-2025-102325 (PMC13084922; doi:10.1136/bmjopen-2025-102325)
Supplement: online supplemental file 1 [file bmjopen-16-4-s001.docx]

## Additional Files:

**Additional File 1**: StaRI and GRAMMS

**Additional File 2:** Conditions included in the definition of multimorbidity

**Additional File 3:** Detailed description of statistical analysis.

**Additional File 4:** Qualitative codes

**Additional File 5**: Description of all adult patients at included practices and the number and percentage who had multimorbidity

**Additional File 6:** Description of eligible and per-protocol patient cohort

**Additional File 7:** NoMAD questionnaire: responses by staff group

**Additional File 8**: NoMAD questionnaire: responses by experience using template

**Additional File 9:** Reach – Initial and / or main review template used (post implementation period: April 2022-June 2023)

**Additional File 10 (a/b):** Fidelity

**Additional File 10a:** All eligible patients (intention to treat analyses)

**Additional File 10b:** Per protocol analyses

## Additional File 1:StaRI and GRAMMS

**
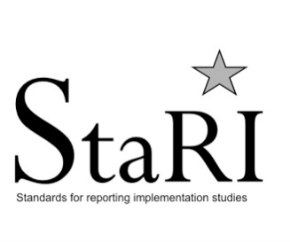
Standards for Reporting Implementation Studies: the StaRI checklist for completion(1)**

| **Checklist item** | | **Reported on page #** | **Implementation Strategy** | **Reported on page #** | **Intervention** |
| --- | --- | --- | --- | --- | --- |
|  | |  | “Implementation strategy” refers to how the intervention was implemented |  | “Intervention” refers to the healthcare or public health intervention that is being implemented. |
| **Title and abstract** | | | | | |
| Title | **1** | 1 | Identification as an implementation study, and description of the methodology in the title and/or keywords | | |
| Abstract | **2** | 3 | Identification as an implementation study, including a description of the implementation strategy to be tested, the evidence-based intervention being implemented, and defining the key implementation and health outcomes. | | |
| **Introduction** | | | | | |
| Introduction | **3** | 6 | Description of the problem, challenge or deficiency in healthcare or public health that the intervention being implemented aims to address. | | |
| Rationale | **4** | Introduction P6-7 and Methods P8-10  Figure 1 and Box 1 | The scientific background and rationale for the implementation strategy (including any underpinning theory/framework/model, how it is expected to achieve its effects and any pilot work). | Methods P9  Figure 1 | The scientific background and rationale for the intervention being implemented (including evidence about its effectiveness and how it is expected to achieve its effects). |
| Aims and objectives | **5** | Methods P7 | The aims of the study, differentiating between implementation objectives and any intervention objectives. | | |
| **Methods: description** | | | | | |
| Design | **6** | Methods P7-8 | The design and key features of the evaluation, (cross referencing to any appropriate methodology reporting standards) and any changes to study protocol, with reasons | | |
| Context | **7** | Methods P8 | The context in which the intervention was implemented. (Consider social, economic, policy, healthcare, organisational barriers and facilitators that might influence implementation elsewhere). | | |
| Targeted ‘sites’ | **8** | Methods P8, 10,11  Results | The characteristics of the targeted ‘site(s)’ (e.g locations/personnel/resources etc.) for implementation and any eligibility criteria. | Methods P10,11 | The population targeted by the intervention and any eligibility criteria. |
| Description | **9** | Methods P9, Box 1 | A description of the implementation strategy | Methods P9 | A description of the intervention |
| Sub-groups | **10** | n/a | Any sub-groups recruited for additional research tasks, and/or nested studies are described | | |
| **Methods: evaluation** | | | | | |
| Outcomes | **11** | Methods P11-13 | Defined pre-specified primary and other outcome(s) of the implementation strategy, and how they were assessed. Document any pre-determined targets | n/a | Defined pre-specified primary and other outcome(s) of the intervention (if assessed), and how they were assessed. Document any pre-determined targets |
| Process evaluation | **12** | Methods P11-13 | Process evaluation objectives and outcomes related to the mechanism by which the strategy is expected to work | | |
| Economic evaluation | **13** | n/a | Methods for resource use, costs, economic outcomes and analysis for the implementation strategy | n/a | Methods for resource use, costs, economic outcomes and analysis for the intervention |
| Sample size | **14** | Methods P10 | Rationale for sample sizes (including sample size calculations, budgetary constraints, practical considerations, data saturation, as appropriate) | | |
| Analysis | **15** | Methods p11-13  Additional File 3 | Methods of analysis (with reasons for that choice) | | |
| Sub-group analyses | **16** | n/a | Any a priori sub-group analyses (e.g. between different sites in a multicentre study, different clinical or demographic populations), and sub-groups recruited to specific nested research tasks | | |

| **Results** | | | | | |
| --- | --- | --- | --- | --- | --- |
| Characteristics | **17** | Results p14-16 | Proportion recruited and characteristics of the recipient population for the implementation strategy | Results p14-16 | Proportion recruited and characteristics (if appropriate) of the recipient population for the intervention |
| Outcomes | **18** | Results p21-23 | Primary and other outcome(s) of the implementation strategy | n/a | Primary and other outcome(s) of the Intervention (if assessed) |
| Process outcomes | **19** | Results p16-26 (qualitative data) | Process data related to the implementation strategy mapped to the mechanism by which the strategy is expected to work | | |
| Economic evaluation | **20** | n/a | Resource use, costs, economic outcomes and analysis for the implementation strategy | n/a | Resource use, costs, economic outcomes and analysis for the intervention |
| Sub-group analyses | **21** | n/a | Representativeness and outcomes of subgroups including those recruited to specific research tasks | | |
| Fidelity/ adaptation | **22** | Main text p21-22 and p23-24 (qualitative data) | Fidelity to implementation strategy as planned and adaptation to suit context and preferences | Main text p21-22 | Fidelity to delivering the core components of intervention (where measured) |
| Contextual changes | **23** | Results P16-19 (qualitative data) | Contextual changes (if any) which may have affected outcomes | | |
| Harms | **24** |  | All important harms or unintended effects in each group | | |
| **Discussion** | | | | | |
| Structured discussion | **25** | P24 | Summary of findings, strengths and limitations, comparisons with other studies, conclusions and implications | | |
| Implications | **26** | P25-26 | Discussion of policy, practice and/or research implications of the implementation strategy (specifically including scalability) | P15-16 | Discussion of policy, practice and/or research implications of the intervention (specifically including sustainability) |
| **General** | | | | | |
| Statements | **27** | Main text p29-29 | Include statement(s) on regulatory approvals (including, as appropriate, ethical approval, confidential use of routine data, governance approval), trial/study registration (availability of protocol), funding and conflicts of interest | | |

GRAMMS checklist(2)

| GRAMMS checklist item | Reported on page # |
| --- | --- |
| Describe the justification for using a mixed methods approach to the research question | Methods P7-8 |
| Describe each method in terms of sampling, data collection and analysis | Methods P8, 10-13 |
| Describe where integration has occurred, how it has occurred and who has participated in it | Methods P14 |
| Describe any limitation of one method associated with the present of the other method | Not explicitly discussed |
| Describe any insights gained from mixing or integrating methods | Discussion p24 onward |

1. Pinnock H, Barwick M, Carpenter CR, Eldridge S, Grandes G, Griffiths CJ, et al. Standards for Reporting Implementation Studies (StaRI) Statement. BMJ. 2017;356:i6795.

2. O'Cathain A, Murphy E, Nicholl J. The quality of mixed methods studies in health services research. J Health Serv Res Policy. 2008;13(2):92-8.

## Additional File 2. Conditions included in the definition of multimorbidity

| The following long-term conditions are included because they benefit from regular review in general practice. Some related conditions (e.g. the first group listed) are grouped so that two or more diagnoses within the group just count as one for the purpose of defining multimorbidity |
| --- |
| - Cardiovascular disease: coronary heart disease, hypertension, heart failure, peripheral arterial disease or chronic kidney disease (stage 3 to 5), Atrial fibrillation |
| - Stroke/TIA |
| - Diabetes |
| - Chronic Obstructive Pulmonary Disease or Asthma |
| - Epilepsy |
| - Depression OR Severe mental health problems (schizophrenia or psychotic illness) |
| - Learning disability |
| - Rheumatoid arthritis |
| - Dementia or Frailty (severe): Although not a single diagnosis, if a patient is on the frailty register it makes sense to do their annual review as part of this annual multimorbidity review, rather than calling the patient back again. |

## Additional File 3: Detailed description of statistical analysis.

Continuous data are summarised as means and standard deviations (SDs), or medians and interquartile ranges (IQRs) if distributions are skewed. Categorical data are summarised as numbers and percentages.

For statistical comparison of fidelity outcomes between intervention and control groups a difference-in-difference framework was used, where the measure of interest was the additional effect of the intervention in the post-intervention period, over and above any effect of period (pre/post) and on intervention group (intervention/ control). For assessment of reach, the measure of interest was the effect of the patient characteristics on template use in the post period in the intervention group only.

For the fidelity outcomes, mixed effect logistic regression models were fitted with intervention (intervention vs. control), time point (pre vs. post-intervention) and the interaction between the two, as well as age group (18-49, 50-59, 60-69, 70-79, and 80+ years), sex, ethnicity (white vs. non-white) and IMD quintile, as fixed effects, and patient ID nested within practice ID as random effects. Odds ratios and corresponding 95% confidence intervals (CIs) and p-values for the interaction term are presented. Some of the numbers were reasonably small and the difference between the groups at baseline were quite large. As such, the logistic regression results were not always intuitive. Therefore, analysis of the percentage of patients with the outcome per practice was also performed on the continuous scale using mixed effect linear regression. Binary intervention and timepoint variables, and the interaction between the two were fitted as fixed effects, along with mean age, mean IMD decile, percentage white and percentage female per practice. Practice ID was fitted as a random effect. Mean differences, and corresponding 95%CIs and p-values are presented.

For the analysis of reach, analyses were restricted to patients from intervention practices in the post-intervention period. Mixed effects logistic regression was used, with the outcome of interest as whether a template (initial and/or main) was used. Age group, sex, ethnicity and IMD quintile were all fitted as fixed effects in the same model; for the long-term conditions, each condition was fitted individually in a model, adjusting for age, sex, ethnicity and IMD as additional fixed effects. Practice ID was fitted as a random effect in all models. Odds ratios, 95%CIs and p-values are presented. For age group and IMD quintile, the p-value for the effect of the variable as a whole, rather than individual p-values for each group, are presented. All p values are two-sided.

For all linear and negative binomial analyses, model fit was explored, and outliers were removed. No adjustments were made for multiple comparisons.

## Additional File 4: Qualitative codes

Example of how the qualitative data (interviews and fieldnotes) were organised under four constructs (Coherence building, Cognitive participation, Collective action, and Reflexive monitoring). ‘Empirical propositions’ derived from the data were grouped (shown below) and themes developed.

| **Implementation Mechanisms** | | |
| --- | --- | --- |
|  | ***Construct*** | ***Theme*** |
|  | Coherence Building | |
|  |  | Understanding of the template / rationale is built through using it |
|  |  | Barriers to understanding |
|  |  | Staff roles and responsibilities |
|  | Cognitive participation | |
|  |  | Buy-in to template because benefit/rationale is clear |
|  |  | Barriers to buying-in to the template |
|  |  | Staff perception of patient’s attitudes |
|  | Collective action | |
|  |  | Admin and recall systems |
|  |  | Buy-in to systems change |
|  |  | Clinic arrangements |
|  |  | Financial incentives |
|  |  | HCAs and receptionists as patient guides |
|  |  | MM reviews can free up other staff |
|  |  | Staff roles |
|  |  | Treating the intervention as a pilot |
|  |  | Normalisation |
|  |  | Process |
|  |  | Patient Benefits |
|  |  | Follow-up appointments and referrals |
|  |  | Scheduling issues |
|  |  | Skills and Training |
|  |  | Template use |
|  |  | Template training |
|  |  | Template and personalised care |
|  |  | Two part reviews |
|  | Reflexive monitoring | |
|  |  | Implementation Process per se |
|  |  | Structure and content of the Arden’s Template |
|  |  | Practical impact of the process of implementing the Arden’s Multimorbidity template |
|  |  | Benefits for staff and patients of implementing the Arden’s multimorbidity template |

## Additional File 5: Description of all adult patients at included practices and the number and percentage who had multimorbidity

|  | |  | | |  | | |
| --- | --- | --- | --- | --- | --- | --- | --- |
|  | | **Implementation practices**  **(n=193,560)** | | | **Control practices**  **(n=203,662)** | | |
|  | | **All adult patients** | **Patients with multimorbidity**  **n, %** | | **All adult patients** | **Patients with multimorbidity**  **n, %** | |
|  | | **N** | **n** | **%** | **N** | **n** | **%** |
| **All patients at included practices** | | 193560 | 8807 | 4.6% | 203662 | 9093 | 4.5% |
| By age groups | |  |  |  |  |  |  |
|  | 18-49 years | 106444 | 678 | 0.6% | 112073 | 719 | 0.6% |
|  | 50-59 years | 30374 | 1257 | 4.1% | 32852 | 1364 | 4.2% |
|  | 60-69 years | 25136 | 1910 | 7.6% | 26854 | 2125 | 7.9% |
|  | 70-79 years | 19597 | 2468 | 12.6% | 20043 | 2411 | 12.0% |
|  | 80+ years | 12009 | 2494 | 20.8% | 11840 | 2474 | 20.9% |
| By sex | |  |  |  |  |  |  |
|  | Male | 96018 | 3951 | 4.1% | 101898 | 3992 | 3.9% |
|  | Female | 97521 | 4856 | 5.0% | 101745 | 5101 | 5.0% |

Note. This table includes all patients meeting the definition of multimorbidity (see supplementary figure 1) and currently registered at the point of data extraction (July 2023). Numbers will therefore not match up to the eligible patients included in the rest of the analyses. The number of patients who were eligible at the point of data extraction but had not been at earlier points in the study periods are described in Figure 1 but not included in any subsequent analyses.

## Additional File 6: Description of eligible and per-protocol patient cohort

|  | | **All eligible patients*** | | | | **Per-protocol patients**** | | | |
| --- | --- | --- | --- | --- | --- | --- | --- | --- | --- |
|  | | **Implementation practices**  **(n=5,060)** | | **Control practices**  **(n=5,363)** | | **Implementation practices**  **(n=2,331)** | | **Control practices**  **(n=2,326)** | |
|  | | **n** | **%** | **n** | **%** | **n** | **%** | **n** | **%** |
| **Demography** | |  |  |  |  |  |  |  |  |
| Age categories | |  |  |  |  |  |  |  |  |
|  | 18-49 years | 291 | 5.8% | 312 | 5.8% | 103 | 4.4% | 150 | 6.4% |
|  | 50-59 years | 704 | 13.9% | 822 | 15.3% | 309 | 13.3% | 364 | 15.6% |
|  | 60-69 years | 1182 | 23.4% | 1304 | 24.3% | 544 | 23.3% | 580 | 24.9% |
|  | 70-79 years | 1514 | 29.9% | 1519 | 28.3% | 756 | 32.4% | 635 | 27.3% |
|  | 80+ years | 1369 | 27.1% | 1406 | 26.2% | 619 | 26.6% | 597 | 25.7% |
| Sex | |  |  |  |  |  |  |  |  |
|  | Male | 2297 | 45.4% | 2364 | 44.1% | 1102 | 47.3% | 1029 | 44.2% |
|  | Female | 2763 | 54.6% | 2999 | 55.9% | 1229 | 52.7% | 1297 | 55.8% |
| Ethnicity | |  |  |  |  |  |  |  |  |
|  | White | 4603 | 91.0% | 4582 | 85.4% | 2142 | 91.9% | 1814 | 78.0% |
|  | Black | 38 | 0.8% | 180 | 3.4% | 17 | 0.7% | 148 | 6.4% |
|  | Asian | 56 | 1.1% | 213 | 4.0% | 17 | 0.7% | 173 | 7.4% |
|  | Mixed | 51 | 1.0% | 45 | 0.8% | 22 | 0.9% | 24 | 1.0% |
|  | Other | 3 | 0.1% | 8 | 0.1% | 2 | 0.1% | 6 | 0.3% |
|  | Missing | 309 | 6.1% | 335 | 6.2% | 131 | 5.6% | 161 | 6.9% |
| IMD quintiles | |  |  |  |  |  |  |  |  |
|  | 1 | 1698 | 33.6% | 1886 | 35.2% | 805 | 34.5% | 979 | 42.1% |
|  | 2 | 958 | 18.9% | 967 | 18.0% | 516 | 22.1% | 422 | 18.1% |
|  | 3 | 933 | 18.4% | 838 | 15.6% | 454 | 19.5% | 385 | 16.6% |
|  | 4 | 874 | 17.3% | 936 | 17.5% | 403 | 17.3% | 291 | 12.5% |
|  | 5 | 594 | 11.7% | 732 | 13.6% | 153 | 6.6% | 247 | 10.6% |
|  | Missing | 3 | 0.1% | 4 | 0.1% | 0 | 0.0% | 2 | 0.1% |
| Number of long-term conditions | |  |  |  |  |  |  |  |  |
|  | 3 | 3326 | 65.7% | 3485 | 65.0% | 1494 | 64.1% | 1463 | 62.9% |
|  | 4 | 1335 | 26.4% | 1482 | 27.6% | 626 | 26.9% | 684 | 29.4% |
|  | 5+ | 399 | 7.9% | 396 | 7.4% | 211 | 9.1% | 179 | 7.7% |
| **Long term conditions:** | |  |  |  |  |  |  |  |  |
| CVD | | 4656 | 92.0% | 4976 | 92.8% | 2186 | 93.8% | 2143 | 92.1% |
|  | CHD | 1225 | 24.2% | 1298 | 24.2% | 607 | 26.0% | 579 | 24.9% |
|  | Atrial fibrillation | 872 | 17.2% | 869 | 16.2% | 436 | 18.7% | 374 | 16.1% |
|  | Hypertension | 3950 | 78.1% | 4254 | 79.3% | 1866 | 80.1% | 1840 | 79.1% |
|  | Heart failure | 614 | 12.1% | 671 | 12.5% | 309 | 13.3% | 305 | 13.1% |
|  | PAD | 357 | 7.1% | 317 | 5.9% | 174 | 7.5% | 121 | 5.2% |
|  | CKD | 1486 | 29.4% | 1630 | 30.4% | 738 | 31.7% | 657 | 28.2% |
| Respiratory disease | | 2725 | 53.9% | 2937 | 54.8% | 1303 | 55.9% | 1256 | 54.0% |
|  | COPD | 1332 | 26.3% | 1455 | 27.1% | 686 | 29.4% | 590 | 25.4% |
|  | Asthma | 1714 | 33.9% | 1858 | 34.6% | 774 | 33.2% | 813 | 35.0% |
| Mental health (SMI or depression) | | 2873 | 56.8% | 2994 | 55.8% | 1182 | 50.7% | 1256 | 54.0% |
|  | Depression | 2737 | 54.1% | 2840 | 53.0% | 1139 | 48.9% | 1183 | 50.9% |
|  | Severe mental illness (QOF) | 284 | 5.6% | 333 | 6.2% | 101 | 4.3% | 145 | 6.2% |
| Dementia or severe frailty | | 1442 | 28.5% | 1355 | 25.3% | 662 | 28.4% | 705 | 30.3% |
|  | Dementia | 414 | 8.2% | 461 | 8.6% | 147 | 6.3% | 192 | 8.3% |
|  | Severe frailty | 1224 | 24.2% | 1094 | 20.4% | 590 | 25.3% | 615 | 26.4% |
| Stroke | | 1470 | 29.1% | 1584 | 29.5% | 686 | 29.4% | 669 | 28.8% |
| Diabetes | | 3176 | 62.8% | 3421 | 63.8% | 1651 | 70.8% | 1513 | 65.0% |
| Epilepsy | | 431 | 8.5% | 536 | 10.0% | 144 | 6.2% | 235 | 10.1% |
| Learning disability register | | 172 | 3.4% | 196 | 3.7% | 57 | 2.4% | 82 | 3.5% |
| Rheumatoid arthritis | | 416 | 8.2% | 425 | 7.9% | 204 | 8.8% | 184 | 7.9% |

* This table only includes patients who were eligible and registered at all three time points (see text). All demographic data are based on the date of data extraction: approximately July-August 2023.

**Per-protocol patients are eligible patients from implementation practices in whom the initial and/or main template had been used in the post-implementation period, and patients from control practices where no eligible patients had ever used the initial or main templates in either time period (eight control practices were included in the per-protocol analyses).

## Additional File 7: NoMAD questionnaire: responses by staff group

|  |  | **Nurse**  **(n=22)** | | **Healthcare Assistant**  **(n=12)** | | **Other^^^**  **(n=22)** | | **Overall**  **(n=56)** | |
| --- | --- | --- | --- | --- | --- | --- | --- | --- | --- |
| **NoMAD questionnaire** | | **Mean** | **Sd** | **Mean** | **Sd** | **Mean** | **Sd** | **Mean** | **sd** |
| When you use the template, how familiar does it feel (0=New, 10=Familiar)?* | | 7.0 | 2.9 | 7.5 | 3.4 | 4.8 | 3.3 | 6.3 | 3.3 |
| Do you feel the template is currently a normal part of your work (0=Not at all, 10=Completely)?* | | 7.3 | 3.1 | 7.9 | 3.1 | 3.7 | 3.7 | 6.1 | 3.8 |
| Do you feel the template will become a normal part of your work (0=Not at all, 10=Completely)?* | | 7.4 | 3.1 | 7.9 | 3.2 | 4.5 | 3.7 | 6.4 | 3.6 |
| **Coherence (1=Strongly agree, 5=Strongly disagree)**** | | 2.1 | 0.6 | 2.0 | 0.7 | 2.9 | 2.0 | 2.4 | 1.4 |
| I can see how the template differs from usual ways of working | |  |  |  |  |  |  |  |  |
|  | Strongly Agree | 3/21 | 14.3% | 2/11 | 18.2% | 3/21 | 14.3% | 8/53 | 15.1% |
|  | Agree | 9/21 | 42.9% | 4/11 | 36.4% | 11/21 | 52.4% | 24/53 | 45.3% |
|  | Neither agree nor disagree | 7/21 | 33.3% | 3/11 | 27.3% | 4/21 | 19.0% | 14/53 | 26.4% |
|  | Disagree | 1/21 | 4.8% | 0/11 | 0.0% | 2/21 | 9.5% | 3/53 | 5.7% |
|  | Strongly disagree | 0/21 | 0.0% | 1/11 | 9.1% | 0/21 | 0.0% | 1/53 | 1.9% |
|  | Don’t know | 1/21 | 4.8% | 1/11 | 9.1% | 1/21 | 4.8% | 3/53 | 5.7% |
| Staff in this organisation have a shared understanding of the purpose of the template | |  |  |  |  |  |  |  |  |
|  | Strongly Agree | 5/21 | 23.8% | 2/11 | 18.2% | 1/21 | 4.8% | 8/53 | 15.1% |
|  | Agree | 12/21 | 57.1% | 6/11 | 54.5% | 12/21 | 57.1% | 30/53 | 56.6% |
|  | Neither agree nor disagree | 4/21 | 19.0% | 2/11 | 18.2% | 5/21 | 23.8% | 11/53 | 20.8% |
|  | Disagree | 0/21 | 0.0% | 0/11 | 0.0% | 2/21 | 9.5% | 2/53 | 3.8% |
|  | Strongly disagree |  |  |  |  |  |  |  |  |
|  | Don’t know | 0/21 | 0.0% | 1/11 | 9.1% | 1/21 | 4.8% | 2/53 | 3.8% |
| I understand how the template affects the nature of my own work | |  |  |  |  |  |  |  |  |
|  | Strongly Agree | 9/21 | 42.9% | 3/9 | 33.3% | 7/20 | 35.0% | 19/50 | 38.0% |
|  | Agree | 8/21 | 38.1% | 4/9 | 44.4% | 6/20 | 30.0% | 18/50 | 36.0% |
|  | Neither agree nor disagree | 2/21 | 9.5% | 1/9 | 11.1% | 5/20 | 25.0% | 8/50 | 16.0% |
|  | Disagree | 2/21 | 9.5% | 1/9 | 11.1% | 0/20 | 0.0% | 3/50 | 6.0% |
|  | Strongly disagree |  |  |  |  |  |  |  |  |
|  | Don’t know | 0/21 | 0.0% | 0/9 | 0.0% | 2/20 | 10.0% | 2/50 | 4.0% |
| I can see the potential value of the template for my work | |  |  |  |  |  |  |  |  |
|  | Strongly Agree | 8/21 | 38.1% | 3/10 | 30.0% | 6/20 | 30.0% | 17/51 | 33.3% |
|  | Agree | 9/21 | 42.9% | 6/10 | 60.0% | 7/20 | 35.0% | 22/51 | 43.1% |
|  | Neither agree nor disagree | 4/21 | 19.0% | 1/10 | 10.0% | 5/20 | 25.0% | 10/51 | 19.6% |
|  | Disagree | 0/21 | 0.0% | 0/10 | 0.0% | 1/20 | 5.0% | 1/51 | 2.0% |
|  | Strongly disagree |  |  |  |  |  |  |  |  |
|  | Don’t know | 0/21 | 0.0% | 0/10 | 0.0% | 1/20 | 5.0% | 1/51 | 2.0% |
| **Cognitive participation (1=Strongly agree, 5=Strongly disagree)***** | | 1.9 | 0.8 | 1.9 | 0.4 | 2.0 | 0.7 | 1.9 | 0.7 |
| There are key people who drive the use of the template forward and get others involved | |  |  |  |  |  |  |  |  |
|  | Strongly Agree | 6/21 | 28.6% | 1/11 | 9.1% | 8/20 | 40.0% | 15/52 | 28.8% |
|  | Agree | 6/21 | 28.6% | 5/11 | 45.5% | 9/20 | 45.0% | 20/52 | 38.5% |
|  | Neither agree nor disagree | 8/21 | 38.1% | 4/11 | 36.4% | 2/20 | 10.0% | 14/52 | 26.9% |
|  | Disagree | 1/21 | 4.8% | 0/11 | 0.0% | 0/20 | 0.0% | 1/52 | 1.9% |
|  | Strongly disagree |  |  |  |  |  |  |  |  |
|  | Don’t know | 0/21 | 0.0% | 1/11 | 9.1% | 1/20 | 5.0% | 2/52 | 3.8% |
| I believe that participating in the use of the template is a legitimate part of my role | |  |  |  |  |  |  |  |  |
|  | Strongly Agree | 8/21 | 38.1% | 3/11 | 27.3% | 5/20 | 25.0% | 16/52 | 30.8% |
|  | Agree | 10/21 | 47.6% | 5/11 | 45.5% | 6/20 | 30.0% | 21/52 | 40.4% |
|  | Neither agree nor disagree | 3/21 | 14.3% | 2/11 | 18.2% | 7/20 | 35.0% | 12/52 | 23.1% |
|  | Disagree | 0/21 | 0.0% | 0/11 | 0.0% | 2/20 | 10.0% | 2/52 | 3.8% |
|  | Strongly disagree |  |  |  |  |  |  |  |  |
|  | Don’t know | 0/21 | 0.0% | 1/11 | 9.1% | 0/20 | 0.0% | 1/52 | 1.9% |
| I'm open to working with colleagues in the new ways to use the template | |  |  |  |  |  |  |  |  |
|  | Strongly Agree | 10/21 | 47.6% | 3/11 | 27.3% | 9/21 | 42.9% | 22/53 | 41.5% |
|  | Agree | 11/21 | 52.4% | 7/11 | 63.6% | 8/21 | 38.1% | 26/53 | 49.1% |
|  | Neither agree nor disagree | 0/21 | 0.0% | 0/11 | 0.0% | 3/21 | 14.3% | 3/53 | 5.7% |
|  | Disagree | 0/21 | 0.0% | 0/11 | 0.0% | 1/21 | 4.8% | 1/53 | 1.9% |
|  | Strongly disagree |  |  |  |  |  |  |  |  |
|  | Don’t know | 0/21 | 0.0% | 1/11 | 9.1% | 0/21 | 0.0% | 1/53 | 1.9% |
| I will continue to support the use of the template | |  |  |  |  |  |  |  |  |
|  | Strongly Agree | 10/21 | 47.6% | 4/11 | 36.4% | 10/21 | 47.6% | 24/53 | 45.3% |
|  | Agree | 9/21 | 42.9% | 6/11 | 54.5% | 7/21 | 33.3% | 22/53 | 41.5% |
|  | Neither agree nor disagree | 1/21 | 4.8% | 0/11 | 0.0% | 4/21 | 19.0% | 5/53 | 9.4% |
|  | Disagree |  |  |  |  |  |  |  |  |
|  | Strongly disagree |  |  |  |  |  |  |  |  |
|  | Don’t know | 1/21 | 4.8% | 1/11 | 9.1% | 0/21 | 0.0% | 2/53 | 3.8% |
| **Collective action (1=Strongly agree, 5=Strongly disagree) ****** | | 2.5 | 0.6 | 2.3 | 0.4 | 2.5 | 1.3 | 2.5 | 0.9 |
| I can easily integrate the template into my existing work | |  |  |  |  |  |  |  |  |
|  | Strongly Agree | 10/21 | 47.6% | 4/11 | 36.4% | 4/20 | 20.0% | 18/52 | 34.6% |
|  | Agree | 9/21 | 42.9% | 5/11 | 45.5% | 5/20 | 25.0% | 19/52 | 36.5% |
|  | Neither agree nor disagree | 2/21 | 9.5% | 1/11 | 9.1% | 6/20 | 30.0% | 9/52 | 17.3% |
|  | Disagree | 0/21 | 0.0% | 0/11 | 0.0% | 2/20 | 10.0% | 2/52 | 3.8% |
|  | Strongly disagree | 0/21 | 0.0% | 0/11 | 0.0% | 2/20 | 10.0% | 2/52 | 3.8% |
|  | Don’t know | 0/21 | 0.0% | 1/11 | 9.1% | 1/20 | 5.0% | 2/52 | 3.8% |
| The template disrupts working relationships | |  |  |  |  |  |  |  |  |
|  | Strongly Agree | 0/21 | 0.0% | 0/11 | 0.0% | 1/20 | 5.0% | 1/52 | 1.9% |
|  | Agree | 0/21 | 0.0% | 1/11 | 9.1% | 4/20 | 20.0% | 5/52 | 9.6% |
|  | Neither agree nor disagree | 5/21 | 23.8% | 3/11 | 27.3% | 3/20 | 15.0% | 11/52 | 21.2% |
|  | Disagree | 9/21 | 42.9% | 4/11 | 36.4% | 10/20 | 50.0% | 23/52 | 44.2% |
|  | Strongly disagree | 7/21 | 33.3% | 1/11 | 9.1% | 1/20 | 5.0% | 9/52 | 17.3% |
|  | Don’t know | 0/21 | 0.0% | 2/11 | 18.2% | 1/20 | 5.0% | 3/52 | 5.8% |
| I have confidence in other people's ability to use the template | |  |  |  |  |  |  |  |  |
|  | Strongly Agree | 4/21 | 19.0% | 1/11 | 9.1% | 6/21 | 28.6% | 11/53 | 20.8% |
|  | Agree | 13/21 | 61.9% | 7/11 | 63.6% | 9/21 | 42.9% | 29/53 | 54.7% |
|  | Neither agree nor disagree | 4/21 | 19.0% | 2/11 | 18.2% | 6/21 | 28.6% | 12/53 | 22.6% |
|  | Disagree |  |  |  |  |  |  |  |  |
|  | Strongly disagree |  |  |  |  |  |  |  |  |
|  | Don’t know | 0/21 | 0.0% | 1/11 | 9.1% | 0/21 | 0.0% | 1/53 | 1.9% |
| Work is assigned to those with skills appropriate to the template | |  |  |  |  |  |  |  |  |
|  | Strongly Agree | 2/21 | 9.5% | 3/11 | 27.3% | 5/20 | 25.0% | 10/52 | 19.2% |
|  | Agree | 16/21 | 76.2% | 6/11 | 54.5% | 12/20 | 60.0% | 34/52 | 65.4% |
|  | Neither agree nor disagree | 3/21 | 14.3% | 1/11 | 9.1% | 2/20 | 10.0% | 6/52 | 11.5% |
|  | Disagree |  |  |  |  |  |  |  |  |
|  | Strongly disagree |  |  |  |  |  |  |  |  |
|  | Don’t know | 0/21 | 0.0% | 1/11 | 9.1% | 1/20 | 5.0% | 2/52 | 3.8% |
| Sufficient training is provided to enable staff to implement the template | |  |  |  |  |  |  |  |  |
|  | Strongly Agree | 5/20 | 25.0% | 2/9 | 22.2% | 6/21 | 28.6% | 13/50 | 26.0% |
|  | Agree | 6/20 | 30.0% | 4/9 | 44.4% | 8/21 | 38.1% | 18/50 | 36.0% |
|  | Neither agree nor disagree | 5/20 | 25.0% | 2/9 | 22.2% | 4/21 | 19.0% | 11/50 | 22.0% |
|  | Disagree | 4/20 | 20.0% | 1/9 | 11.1% | 3/21 | 14.3% | 8/50 | 16.0% |
|  | Strongly disagree |  |  |  |  |  |  |  |  |
|  | Don’t know |  |  |  |  |  |  |  |  |
| Sufficient resources are available to support the use of the template | |  |  |  |  |  |  |  |  |
|  | Strongly Agree | 4/21 | 19.0% | 1/9 | 11.1% | 6/21 | 28.6% | 11/51 | 21.6% |
|  | Agree | 5/21 | 23.8% | 5/9 | 55.6% | 6/21 | 28.6% | 16/51 | 31.4% |
|  | Neither agree nor disagree | 7/21 | 33.3% | 2/9 | 22.2% | 7/21 | 33.3% | 16/51 | 31.4% |
|  | Disagree | 3/21 | 14.3% | 1/9 | 11.1% | 2/21 | 9.5% | 6/51 | 11.8% |
|  | Strongly disagree |  |  |  |  |  |  |  |  |
|  | Don’t know | 2/21 | 9.5% | 0/9 | 0.0% | 0/21 | 0.0% | 2/51 | 3.9% |
| Management adequately supports the use of the template | |  |  |  |  |  |  |  |  |
|  | Strongly Agree | 7/21 | 33.3% | 2/9 | 22.2% | 8/21 | 38.1% | 17/51 | 33.3% |
|  | Agree | 10/21 | 47.6% | 6/9 | 66.7% | 9/21 | 42.9% | 25/51 | 49.0% |
|  | Neither agree nor disagree | 4/21 | 19.0% | 1/9 | 11.1% | 4/21 | 19.0% | 9/51 | 17.6% |
|  | Disagree |  |  |  |  |  |  |  |  |
|  | Strongly disagree |  |  |  |  |  |  |  |  |
|  | Don’t know |  |  |  |  |  |  |  |  |
| **Reflexive monitoring (1=Strongly agree, 5=Strongly disagree) ******* | | 2.6 | 1.1 | 2.2 | 0.4 | 2.9 | 1.7 | 2.6 | 1.3 |
| I am aware of feedback about the effects of the template | |  |  |  |  |  |  |  |  |
|  | Strongly Agree | 3/21 | 14.3% | 0/10 | 0.0% | 4/21 | 19.0% | 7/52 | 13.5% |
|  | Agree | 3/21 | 14.3% | 3/10 | 30.0% | 6/21 | 28.6% | 12/52 | 23.1% |
|  | Neither agree nor disagree | 6/21 | 28.6% | 6/10 | 60.0% | 6/21 | 28.6% | 18/52 | 34.6% |
|  | Disagree | 7/21 | 33.3% | 1/10 | 10.0% | 1/21 | 4.8% | 9/52 | 17.3% |
|  | Strongly disagree |  |  |  |  |  |  |  |  |
|  | Don’t know | 2/21 | 9.5% | 0/10 | 0.0% | 4/21 | 19.0% | 6/52 | 11.5% |
| The staff agree that the template is worthwhile | |  |  |  |  |  |  |  |  |
|  | Strongly Agree | 4/21 | 19.0% | 2/10 | 20.0% | 4/20 | 20.0% | 10/51 | 19.6% |
|  | Agree | 9/21 | 42.9% | 6/10 | 60.0% | 3/20 | 15.0% | 18/51 | 35.3% |
|  | Neither agree nor disagree | 5/21 | 23.8% | 2/10 | 20.0% | 10/20 | 50.0% | 17/51 | 33.3% |
|  | Disagree | 2/21 | 9.5% | 0/10 | 0.0% | 1/20 | 5.0% | 3/51 | 5.9% |
|  | Strongly disagree |  |  |  |  |  |  |  |  |
|  | Don’t know | 1/21 | 4.8% | 0/10 | 0.0% | 2/20 | 10.0% | 3/51 | 5.9% |
| I value the effects that the template has had on my work | |  |  |  |  |  |  |  |  |
|  | Strongly Agree | 6/21 | 28.6% | 2/10 | 20.0% | 6/19 | 31.6% | 14/50 | 28.0% |
|  | Agree | 9/21 | 42.9% | 6/10 | 60.0% | 5/19 | 26.3% | 20/50 | 40.0% |
|  | Neither agree nor disagree | 4/21 | 19.0% | 1/10 | 10.0% | 6/19 | 31.6% | 11/50 | 22.0% |
|  | Disagree | 2/21 | 9.5% | 1/10 | 10.0% | 0/19 | 0.0% | 3/50 | 6.0% |
|  | Strongly disagree | 0/21 | 0.0% | 0/10 | 0.0% | 1/19 | 5.3% | 1/50 | 2.0% |
|  | Don’t know | 0/21 | 0.0% | 0/10 | 0.0% | 1/19 | 5.3% | 1/50 | 2.0% |
| Feedback about the template can be used to improve it in the future | |  |  |  |  |  |  |  |  |
|  | Strongly Agree | 6/21 | 28.6% | 3/10 | 30.0% | 10/21 | 47.6% | 19/52 | 36.5% |
|  | Agree | 12/21 | 57.1% | 7/10 | 70.0% | 8/21 | 38.1% | 27/52 | 51.9% |
|  | Neither agree nor disagree | 3/21 | 14.3% | 0/10 | 0.0% | 3/21 | 14.3% | 6/52 | 11.5% |
|  | Disagree |  |  |  |  |  |  |  |  |
|  | Strongly disagree |  |  |  |  |  |  |  |  |
|  | Don’t know |  |  |  |  |  |  |  |  |
| I can modify how I work with the template | |  |  |  |  |  |  |  |  |
|  | Strongly Agree | 4/21 | 19.0% | 1/10 | 10.0% | 6/20 | 30.0% | 11/51 | 21.6% |
|  | Agree | 9/21 | 42.9% | 5/10 | 50.0% | 5/20 | 25.0% | 19/51 | 37.3% |
|  | Neither agree nor disagree | 4/21 | 19.0% | 4/10 | 40.0% | 7/20 | 35.0% | 15/51 | 29.4% |
|  | Disagree | 3/21 | 14.3% | 0/10 | 0.0% | 1/20 | 5.0% | 4/51 | 7.8% |
|  | Strongly disagree |  |  |  |  |  |  |  |  |
|  | Don’t know | 1/21 | 4.8% | 0/10 | 0.0% | 1/20 | 5.0% | 2/51 | 3.9% |

^^^ GP (n=5), Pharmacist (n=2), Admin/ reception/ secretary (n=3), practice manager/ deputy manager/ operations manager (n=8), trainee nurse (n=1), data coordinator (n=1), data team lead (n=1), clinical research lead (n=1).

Missing data (Nurse, HCA, Other): *(1, 1, 3), **(1, 1, 1), ***(1, 2, 2), ****(2, 3, 2), *****(1, 2, 3)

## Additional File 8: NoMAD questionnaire: responses experience using template

|  | **Not at all**  **(n=14)** | | **1-4 times**  **(n=5)** | | **5-9 times**  **(n=2)** | | **10-15 times**  **(n=6)** | | **More than 15 times**  **(n=28)** | | **Overall**  **(n=55)** | |
| --- | --- | --- | --- | --- | --- | --- | --- | --- | --- | --- | --- | --- |
| **NoMAD questionnaire** | **Mean** | **Sd** | **Mean** | **Sd** | **Mean** | **Sd** | **Mean** | **Sd** | **Mean** | **Sd** | **Mean** | **Sd** |
| When you use the template, how familiar does it feel (0=New, 10=Familiar)? n=50 | 2.4 | 2.9 | 2.5 | 2.0 | 5.0 | 4.2 | 7.2 | 1.3 | 8.3 | 1.9 | 6.3 | 3.3 |
| Do you feel the template is currently a normal part of your work (0=Not at all, 10=Completely)? n=50 | 1.4 | 2.1 | 1.8 | 2.8 | 4.5 | 4.9 | 5.3 | 2.6 | 8.8 | 1.6 | 6.1 | 3.8 |
| Do you feel the template will become a normal part of your work (0=Not at all, 10=Completely)? n=50 | 2.9 | 3.6 | 2.5 | 3.3 | 5.0 | 5.7 | 6.7 | 2.6 | 8.5 | 2.0 | 6.4 | 3.6 |
| **Coherence*** n=49 | 2.9 | 2.0 | 3.9 | 2.4 | 2.1 | 0.5 | 2.0 | 0.5 | 2.0 | 0.6 | 2.4 | 1.4 |
| **Cognitive participation*** n=50 | 1.9 | 0.7 | 2.1 | 0.6 | 2.0 | 1.4 | 2.5 | 1.0 | 1.7 | 0.5 | 1.9 | 0.7 |
| **Collective action*** n=48 | 2.8 | 1.8 | 2.5 | 0.7 | 2.6 | 0.4 | 2.7 | 0.7 | 2.2 | 0.5 | 2.5 | 0.9 |
| **Reflexive monitoring*** n=49 | 2.8 | 1.6 | 3.6 | 1.8 | 3.6 |  | 2.1 | 0.8 | 2.4 | 1.0 | 2.6 | 1.3 |
| *Figures are means for the questions included within this scale, each scored from 1=strongly agree to 5= strongly disagree | | | | | | | | | | | | |

## Additional File 9: Reach – Initial and/or main review template used (post implementation period: April 2022-June 2023)

|  | **All implementation practices**  **(n=5060)** | | **Control practices**  **(n=5363)** | | **Predictors of template use in implementation practices*** | |
| --- | --- | --- | --- | --- | --- | --- |
|  | **n** | **%** | **n** | **%** | **Odds ratio (95% CI)** | **p-value** |
| **Template use:** |  |  |  |  |  |  |
| Neither | 2729/5060 | 53.9% | 4525/5363 | 84.4% |  |  |
| Initial only | 220/5060 | 4.3% | 230/5363 | 4.3% |  |  |
| Main only | 1855/5060 | 36.7% | 602/5363 | 11.2% |  |  |
| Both | 256/5060 | 5.1% | 6/5363 | 0.1% |  |  |
| **Eligible patients who used initial and/or main review template:** | 2331/5060 | 46.1% | 838/5363 | 15.6% |  |  |
| Age categories |  |  |  |  |  | <0.001 |
| 18-49 years | 103/291 | 35.4% | 24/312 | 7.7% | 0.54 (0.39 to 0.74) |  |
| 50-59 years | 309/704 | 43.9% | 114/822 | 13.9% | 0.72 (0.57 to 0.92) |  |
| 60-69 years | 544/1182 | 46.0% | 214/1304 | 16.4% | 0.98 (0.80 to 1.19) |  |
| 70-79 years | 756/1514 | 49.9% | 275/1519 | 18.1% | 1.18 (0.97 to 1.42) |  |
| 80+ years | 619/1369 | 45.2% | 211/1406 | 15.0% | 1 |  |
| Sex |  |  |  |  |  | 0.008 |
| Male | 1102/2297 | 48.0% | 389/2364 | 16.5% | 1 |  |
| Female | 1229/2763 | 44.5% | 449/2999 | 15.0% | 0.83 (0.72 to 0.95) |  |
| Ethnicity |  |  |  |  |  | 0.834 |
| White | 2142/4603 | 46.5% | 749/4582 | 16.3% | 1 |  |
| Black** | 17/38 | 44.7% | 23/180 | 12.8% | 1.05 (0.69 to 1.58)** |  |
| Asian** | 17/56 | 30.4% | 15/213 | 7.0% | N/A** |  |
| Mixed/Other** | 24/54 | 44.4% | 9/53 | 17.0% | N/A** |  |
| IMD quintiles |  |  |  |  |  | 0.010 |
| 1 | 805/1698 | 47.4% | 294/1886 | 15.6% | 1 |  |
| 2 | 516/958 | 53.9% | 242/967 | 25.0% | 1.40 (1.13 to 1.74) |  |
| 3 | 454/933 | 48.7% | 80/838 | 9.5% | 1.12 (0.90 to 1.40) |  |
| 4 | 403/874 | 46.1% | 147/936 | 15.7% | 1.18 (0.94 to .49) |  |
| 5 | 153/594 | 25.8% | 74/732 | 10.1% | 0.89 (0.67 to 1.20) |  |
| Long term conditions |  |  |  |  |  |  |
| CVD | 2186/4656 | 47.0% | 796/4976 | 16.0% | 1.75 (1.34 to 2.30) | <0.001 |
| CHD | 607/1225 | 49.6% | 206/1298 | 15.9% | 1.12 (0.94 to 1.33) | 0.198 |
| Atrial fibrillation | 436/872 | 50.0% | 133/869 | 15.3% | 1.31 (1.07 to 1.59) | 0.009 |
| Hypertension | 1866/3950 | 47.2% | 698/4254 | 16.4% | 1.40 (1.18 to 1.67) | <0.001 |
| Heart failure | 309/614 | 50.3% | 119/671 | 17.7% | 1.28 (1.02 to 1.60) | 0.036 |
| PAD | 174/357 | 48.7% | 68/317 | 21.5% | 0.96 (0.73 to 1.27) | 0.784 |
| CKD | 738/1486 | 49.7% | 305/1630 | 18.7% | 1.29 (1.08 to 1.54) | 0.004 |
| Respiratory disease | 1303/2725 | 47.8% | 469/2937 | 16.0% | 1.11 (0.96 to 1.29) | 0.145 |
| COPD | 686/1332 | 51.5% | 273/1455 | 18.8% | 1.14 (0.97 to 1.34) | 0.120 |
| Asthma | 774/1714 | 45.2% | 274/1858 | 14.7% | 1.05 (0.90 to 1.22) | 0.559 |
| Depression | 1139/2737 | 41.6% | 404/2840 | 14.2% | 0.62 (0.53 to 0.72) | <0.001 |
| Severe mental health problems | 101/284 | 35.6% | 45/333 | 13.5% | 0.69 (0.51 to 0.95) | 0.022 |
| Dementia | 147/414 | 35.5% | 55/461 | 11.9% | 0.54 (0.41 to 0.72) | <0.001 |
| Severe frailty | 590/1224 | 48.2% | 107/1094 | 9.8% | 1.22 (1.00 to 1.50) | 0.051 |
| Stroke | 686/1470 | 46.7% | 258/1584 | 16.3% | 0.97 (0.83 to 1.15) | 0.755 |
| Diabetes | 1651/3176 | 52.0% | 644/3421 | 18.8% | 2.46 (2.11 to 2.87) | <0.001 |
| Epilepsy | 144/431 | 33.4% | 66/536 | 12.3% | 0.47 (0.36 to 0.61) | <0.001 |
| Learning disability | 57/172 | 33.1% | 21/196 | 10.7% | 0.66 (0.43 to 1.00 | 0.049 |
| Rheumatoid arthritis | 204/416 | 49.0% | 55/425 | 12.9% | 1.14 (0.88 to 1.48) | 0.305 |

* This analysis shows the characteristics of patients in whom the template was used in intervention practices in the post-intervention period (not a comparison between intervention and control practices). The odds ratio shows the odds of the template being used in patients with each characteristic compared with patients without this characteristic. See Additional file 3 for details of the statistical analysis.

** Combined non-white ethnic groups because of small numbers

## Additional File 10 (a,b):Fidelity

### Additional File 10(a): All eligible patients (intention to treat analyses):

|  | **All intervention practices**  **Pre-period**  **(n=5060)** | | **All intervention practices**  **Post-period**  **(n=5060)** | | **Control practices**  **Pre-period**  **(n=5363)** | | **Control practices**  **Post-period**  **(n=5363)** | |
| --- | --- | --- | --- | --- | --- | --- | --- | --- |
|  | **n** | **%** | **n** | **%** | **n** | **%** | **n** | **%** |
| **Initial and/or main template use** | 1095 | 21.6% | 2331 | 46.1% | 571 | 10.6% | 838 | 15.6% |
| Mobility assessed | 260 | 5.1% | 759 | 15.0% | 333 | 6.2% | 362 | 6.7% |
| Activities of daily life assessed | 48 | 0.9% | 326 | 6.4% | 6 | 0.1% | 24 | 0.4% |
| Mood assessed | 92 | 1.8% | 113 | 2.2% | 275 | 5.1% | 285 | 5.3% |
| Memory assessed | 139 | 2.7% | 386 | 7.6% | 160 | 3.0% | 185 | 3.4% |
| Falls assessed | 501 | 9.9% | 927 | 18.3% | 827 | 15.4% | 934 | 17.4% |
| Frailty assessed | 800 | 15.8% | 1230 | 24.3% | 542 | 10.1% | 1096 | 20.4% |
| Pain assessed | 151 | 3.0% | 513 | 10.1% | 155 | 2.9% | 223 | 4.2% |
| Medication adherence raised | 819 | 16.2% | 1377 | 27.2% | 701 | 13.1% | 1099 | 20.5% |
| Medication reviewed | 4009 | 79.2% | 4016 | 79.4% | 3973 | 74.1% | 4229 | 78.9% |
| Referred for social prescribing | 397 | 7.8% | 706 | 14.0% | 434 | 8.1% | 888 | 16.6% |
| At least one patient goal identified | 48 | 0.9% | 67 | 1.3% | 66 | 1.2% | 68 | 1.3% |
| Sent patient preparation document | 0 | 0.0% | 247 | 4.9% | 0 | 0.0% | 0 | 0.0% |
| Care and support plan offered | 117 | 2.3% | 219 | 4.3% | 436 | 8.1% | 239 | 4.5% |
| Care and support plan agreed or reviewed | 79 | 1.6% | 280 | 5.5% | 388 | 7.2% | 685 | 12.8% |
| Care and support plan given to patient | 4 | 0.1% | 42 | 0.8% | 4 | 0.1% | 6 | 0.1% |
| Annual review completed | 25 | 0.5% | 664 | 13.1% | 1 | 0.0% | 1 | 0.0% |

### Additional File 10b: Per protocol analyses*

|  | **All intervention practices**  **Pre-period**  **(n=2331)** | | **All intervention practices**  **Post-period**  **(n=2331)** | | **Control practices**  **Pre-period**  **(n=2326)** | | **Control practices**  **Post-period**  **(n=2326)** | |
| --- | --- | --- | --- | --- | --- | --- | --- | --- |
|  | **n** | **%** | **n** | **%** | **n** | **%** | **n** | **%** |
| **Initial and/or main template use** | 875 | 37.5% | 2331 | 100% | 0 | 0.0% | 0 | 0.0% |
| Mobility assessed | 146 | 6.3% | 580 | 24.9% | 221 | 9.5% | 235 | 10.1% |
| Activities of daily life assessed | 35 | 1.5% | 302 | 13.0% | 0 | 0.0% | 2 | 0.1% |
| Mood assessed | 48 | 2.1% | 71 | 3.0% | 233 | 10.0% | 240 | 10.3% |
| Memory assessed | 95 | 4.1% | 325 | 13.9% | 17 | 0.7% | 49 | 2.1% |
| Falls assessed | 225 | 9.7% | 561 | 24.1% | 503 | 21.6% | 516 | 22.2% |
| Frailty assessed | 425 | 18.2% | 675 | 29.0% | 317 | 13.6% | 730 | 31.4% |
| Pain assessed | 76 | 3.3% | 407 | 17.5% | 85 | 3.7% | 83 | 3.6% |
| Medication adherence raised | 508 | 21.8% | 1011 | 43.4% | 358 | 15.4% | 561 | 24.1% |
| Medication reviewed | 1962 | 84.2% | 2005 | 86.0% | 1549 | 66.6% | 1755 | 75.5% |
| Referred for social prescribing | 168 | 7.2% | 313 | 13.4% | 209 | 9.0% | 380 | 16.3% |
| At least one patient goal identified | 16 | 0.7% | 55 | 2.4% | 41 | 1.8% | 23 | 1.0% |
| Sent patient preparation document | 0 | 0.0% | 164 | 7.0% | 0 | 0.0% | 0 | 0.0% |
| Care and support plan offered | 79 | 3.4% | 133 | 5.7% | 117 | 5.0% | 71 | 3.1% |
| Care and support plan agreed or reviewed | 37 | 1.6% | 175 | 7.5% | 255 | 11.0% | 399 | 17.2% |
| Care and support plan given to patient | 4 | 0.2% | 40 | 1.7% | 0 | 0.0% | 0 | 0.0% |
| Annual review completed | 16 | 0.7% | 576 | 24.7% | 0 | 0.0% | 1 | 0.0% |

* Per-protocol patients are eligible patients from implementation practices in whom the initial and/or main template had been used in the post-implementation period, and patients from control practices where no eligible patients had ever used the initial or main templates in either time period (eight control practices were included in the per-protocol analyses).
