# Supplementary material for: Implementation of a comprehensive template to support personalised care for people with multiple long-term conditions: a mixed-methods evaluation in primary care
Source: BMJ Open. 2026 Apr 15;16(4):e102325. doi: 10.1136/bmjopen-2025-102325 (PMC13084922; doi:10.1136/bmjopen-2025-102325)
Supplement: Supplementary file 2 [file bmjopen-2025-102325.reviewer_comments.pdf]

PEER REVIEW HISTORY

BMJ Open publishes all reviews undertaken for accepted manuscripts. Reviewers are asked to complete a checklist review form and are provided with free text boxes to elaborate on their assessment. These free text comments are reproduced below.

ARTICLE DETAILS

Title (Provisional)

Implementation of a comprehensive template to support personalised care for people with multiple long-term conditions: A mixed-methods evaluation in primary care

Authors

Johnson, Rachel; Turner, Andrew; Jinks, Clare; Portillo , Mari Carmen; Coope, Caroline; Moult, Alice; Lippiett, Kate Alice; Baker, Dereth June June; Mann, Cindy; Scott, Lauren J; Dziedzic, Krysia; Paskins, Zoe; Byng, Richard; Chilcott, Simon; Scrimgeour, Grace; Salisbury, Chris

---

VERSION 1 - REVIEW

Reviewer 1

Name Elvey, Rebecca

Affiliation University of Manchester Institute of Population Health, Centre for Primary Care

Date 07-Oct-2025

COI None

The manuscript reports an interesting study, with application of NPT to the implementation of a new template in primary care, comparing practices with and without an implementation package. I have made both broader and more specific comments and questions below.

What were the main areas of questionning in the interviews?

With regards to the application of the NPT coding manual for the qualitative data, what were the results of coding deductively to the 12 NPT constructs? - as apart from the four main constructs, the results do not seem to refer to these.

The analysis process seems to have discerned many factors relevant to implementation, as I read through the paper, more detail on some of these would have been useful, and I think could bring the findings to life more, however, with so many different factors discussed I then ended up unsure about what was most important. For example, the sentence at line 390 mentions practice-level champions, but I wasn't sure who these were or what they contributed. The discussion needs to relate more clearly to the results presented (and my

comments here relate to my response to question 11 above, hopefully addressing some of these would draw out the key findings more clearly). For example, the discussion point starting at line 565 refers to impactful strategy components and the paragraph goes on to mention process mapping, but this is not reflected in the findings. The paper does bring together qualitative and quantitative findings, but it wasn't always clear how these worked together. There were quantitative findings that seemed really important, in particular, that the 'initial consultation' template was much less used and also differences in use of the templates between patients of different age, areas with different deprivation levels and patients with certain conditions - did interviews ask about these or generate data on them?

Regarding my negative response to question 8 above: The introduction would benefit from references to locate the paper within the evidence base on the implementation of interventions aimed at improving personalised care, for multimorbidity, in primary care, or, if this literature does not exist, acknowledgement of this.

Line 242 - routine data - what does this refer to? Which data were collected? and why?

Terminology such as 'deprivation decile' (line 258) needs explanation, especially for an international audience. Also, line 261 - 'lists of codes' needs explanation.

Line 289 - regarding the qualitative sampling strategy and use of purposive sampling - were you aiming to look for/ascertain differences between practices and between types of staff role in the ways the template was implemented? Could you explain further e.g. what were the different ways of implementing the template - and how did you know how this was being done?

Qualitative interviews: How were participants selected and approached? Over what time period did the interviews take place? I think each participant was interviewed once, but the section starting at line 504 seems to refer to staff views changing over time, please could you clarify?

The sentence starting in line 298 seems incomplete.

Discussion:

Line 524 - sentence beginning 'Patients with multimorbidity' - I didn't understand this sentence.

Line 545 - why would the practices recruited first have more motivation that control practices?

**Reviewer**	**2**
**Name**	**Kelson, Zoe**
**Affiliation**	**University of Exeter, Mathematics**
**Date**	**19-Nov-2025**

COI **None**

This study seeks to understand and address the challenges of implementing a template to support personalised primary care for people with multimorbidity (PP4M).

Reviewer comments:

"This paper evaluates implementation of the PP4M template from the perspective of general practice and is reported using STARi(16) and GRAMMS(17) checklists (see Additional File 1). "

Thanks for providing a copy of the associated checklists.

"Primary care (GP practices) in three English regions" [Abstract]

and

"Practices in deprived areas were prioritised, because multimorbidity is more prevalent in these areas.(5) Template implementation occurred in 2022-3.(18) "

and

"No formal sample size calculation was made. Intervention and control practices were recruited with the intention of obtaining wide variation across practices in practice size, urban/rural location, and deprivation and ethnicity of patient populations"

and

"To compare implementation and control practices, we included patients who were registered at the practice at all three time points (1.1.21, 249 1.4.22, 30.6.23). "

Can the authors please comment on whether they consider the included patients and practices to be representative?

Can the authors please consider applying sample weights to adjust for the selection process of practices?

"Routine medical record data; NoMAD questionnaires and qualitative interviews in implementation practices"

and

"Age, sex, deprivation decile, ethnicity, and number and type of long-term conditions were extracted at patient level for all eligible patients"

Can the authors please confirm if there was any missing data?

" Quantitative data collection included the Normalisation MeAsure Development (NoMAD) questionnaire,(21) a 23-item survey for assessing implementation of complex interventions in healthcare from the perspective of professionals directly involved"

Can the authors please comment on whether they may be potential for self-reporting bias?

"Normalisation Process Theory (NPT) seeks to understand how innovations become normalised in 174 healthcare settings"

and

"For qualitative analysis, we used May et al.'s NPT coding manual which combines NPT with the Context Mechanism-Outcome (CMO) configuration of realist evaluation studies...

In line with the NPT coding manual, our initial logic model (Figure 1) described the contextual factors we considered when developing the implementation strategy and intervention, the mechanisms by which both were anticipated to act, and the outcomes by which each would be evaluated. "

The qualitative approach is clearly described.

"Data analysis is summarised below and described in detail in Additional File 3. "

and

"Additional File 3: Detailed description of statistical analysis"

Can more detail please be moved into the main article?

"assessed in the implementation period in implementation sites at the patient level using multilevel logistic regression with practice as a random effect, and adjusted for demographics (age, sex, ethnicity and IMD) as fixed effects. Further models added each long-term condition in turn, to assess differences in template use by condition. "

and

"Using multilevel logistic regression we assessed whether use of personalised care sections of the template differed between pre and implementation periods, adjusting for control practices, and between intervention and control practices, in a difference-in-difference framework. Models were adjusted for demographics (as above) as fixed effects, and patient ID nested with practice ID as random effects. Fidelity was further modelled at the practice level as the percentage of patients at each practice with the outcome. "

Appropriate modelling methods have been applied.

"Quantitative and qualitative results were considered side-by-side; the extent to which the data converged /diverged was discussed with analytic insights developed through discussion. "

Can the authors please consider performing a Delphi process to reach a consensus?

"Fifty-six staff from 13 practices completed the NoMAD questionnaire. Agreement with statements about coherence was generally high, particularly among nurses and healthcare assistants (Additional File 7)"

Whilst no formal sample size calculation was conducted, can the authors please discuss sample size in the study limitations?

"The patients in whom the template was used were broadly similar to the cohort of all eligible patients (Additional File 6)."

and

"It was less well used in younger patients, those in least deprived areas and in eligible patients with epilepsy, mental health problems, dementia or learning difficulties, but more likely to be used in patients with CVD or with diabetes (Additional File 9)."

and

"There was a clear trend of greater familiarity with greater use of the template"

Can statistical outcomes please be reported in support of study findings throughout the Results text (n.b. the above excerpts are for example only)?

"Because this is a non-randomised study, there could be differences between implementation and control practices which impact on our conclusions" [Strengths and Limitations of this study]

Can the Discussion of the study limitations please be expanded on?

Thanks for providing a copy of the protocol.

VERSION 1 - AUTHOR RESPONSE

	Reviewer 1<br>Dr. Rebecca Elvey, University of Manchester Institute of Population Health	
1.1	What were the main areas of questioning in the interviews?	We have added a description of the content of the topic guide to the first paragraph of the section 'Qualitative data collection and analysis', as follows:<br><br>"The topic guide explored: (i) how the new review process operated within the practice, including workflow changes, benefits, challenges, and impacts; and (ii) for those directly conducting reviews, how they used the review template, its usability, and its perceived influence on the conduct and quality of patient reviews."
1.2	With regards to the application of the NPT coding manual for the qualitative data, what were the results of coding deductively to the 12 NPT constructs?	As described in the section 'Qualitative data collection and analysis' the qualitative data was coded deductively to all 12 NPT

	- as apart from the four main constructs, the results do not seem to refer to these.	constructs, and analysed as described in that section (see below). In line with the NPT coding manual, data relating to the four main NPT constructs is presented within the 'Mechanisms' section of the results. Themes developed from the data coded to the four constructs relating to 'Context' and the four constructs related to 'Outcomes' are presented in the 'Context' and 'Outcomes' sections of the results respectively. We have added text to the 'Qualitative data collection and analysis' section to clarify this.<br><br>The section 'Qualitative data collection and analysis' now reads: (additional text highlighted)<br><br>''The twelve primary constructs of the NPT coding manual were used for analysis.(22) Researchers familiarised themselves with transcripts and the NPT constructs and met regularly to ensure consistent use of the codes, and to discuss interpretation of the data. Data were coded deductively to the twelve NPT constructs, with additional inductively developed codes (abductive coding). Data for each code were summarised into a set of summary propositions (statements describing the developing findings), with accompanying quotes. Fieldnotes were read to identify evidence to support propositions or suggest new propositions. Propositions were grouped, and themes developed, and through team discussion, including with the public collaborators, the key messages were developed. Findings related to the four main NPT constructs are reported within the 'Mechanisms' section of the results. Themes developed from the data coded to the four constructs relating to 'Context' and the four constructs related to 'Outcomes' are presented in the 'Context' and 'Outcomes' sections of the results respectively.''

1.3	The analysis process seems to have discerned many factors relevant to implementation, as I read through the paper, more detail on some of these would have been useful, and I think could bring the findings to life more, however, with so many different factors discussed I then ended up unsure about what was most important. For example, the sentence at line 390 mentions practice-level champions, but I wasn't sure who these were or what they contributed.	Within the constraints of the word count for a mixed methods study, we have sought to describe the breadth of factors relevant to implementation, rather than give detailed information about a smaller number. In this study, our methods did not enable us to link individual factors to specific outcomes of implementation.<br><br>Regarding practice-level champions. There was no formal 'champion' role in the study. However, in some practices there were practice staff who were engaged and enthusiastic supporters of the intervention.<br><br>We have replaced 'practice-level champions of the intervention' with 'engaged practice staff who supported use of the intervention'.<br><br>The sentence now reads:<br>"Having engaged practice staff who supported use of the intervention, protected time for supporting change processes, and involving the wider staff team in planning changes increased buy-in and supported implementation"
1.4	The discussion needs to relate more clearly to the results presented (and my comments here relate to my response to question 11 above, hopefully addressing some of these would draw out the key findings more clearly). For example, the discussion point starting at line 565 refers to impactful strategy components and the paragraph goes on to mention process mapping, but this is not reflected in the findings. The paper does bring together qualitative and quantitative findings, but it wasn't always clear how these worked together. There were quantitative findings that seemed really important,	Thankyou for this comment. We have edited the discussion to better link it with the findings<br><br>**Re: impactful strategy components and process mapping.**<br>We did find that process mapping, a component of our implementation strategy as described in the methods, supported implementation. We have made this more clear in the results section, with additional text in lines 412 and 440.<br><br>In the discussion, we have clarified the point about impactful strategy

	in particular, that the 'initial consultation' template was much less used and also differences in use of the templates between patients of different age, areas with different deprivation levels and patients with certain conditions - did interviews ask about these or generate data on them?	components – the reworded paragraph now reads:<br><br>"In this study, the four core constructs of NPT were confirmed as mechanisms supporting implementation. In our implementation strategy, process mapping, and ongoing interactions with researchers / research facilitators supported these mechanisms. By providing the opportunity to engage in discussion about existing practice processes and the fit of the intervention with practice processes, these implementation strategy components helped practice staff to understand, individually and collectively, how the template worked, and how it could be used in practice within an individual or team's existing work practices, i.e. supporting coherence and cognitive participation."<br><br>**Re: relationship of quantitative and qualitative findings**<br>This was a parallel mixed methods study, and the quantitative and qualitative data collection were concurrent. Therefore, we did not use the qualitative study to ask about / generate data on the quantitative study findings. However, to address your comment, we have added to the discussion of our findings, as below:<br><br>"The template was less well used in younger patients. This may reflect lower numbers of eligible younger patients, or practices choosing to prioritise comprehensive reviews for older people. We found that the template was more likely to be used in patients with CVD or diabetes, and less likely to be used in patients with epilepsy, mental health problems,

		dementia or learning difficulties. It is likely that this is because CVD and diabetes are common conditions in which nurses develop advanced skills, whereas fewer primary care practices have nurses with advanced skills in epilepsy, mental health, dementia or learning difficulties. In addition, practices may have existing mechanisms for reviewing patients with some conditions, for example annual reviews for people with learning difficulties are incentivised by the QOF. The initial consultation template was much less used than the annual review template. This may reflect lack of resources to deliver, or perceived inefficiency of, two separate appointments."
1.5	Regarding my negative response to question 8 above: The introduction would benefit from references to locate the paper within the evidence base on the implementation of interventions aimed at improving personalised care, for multimorbidity, in primary care, or, if this literature does not exist, acknowledgement of this.	Thankyou, we have added an additional short paragraph in the introduction locating the paper within this evidence base.
1.6	Line 242 - routine data - what does this refer to? Which data were collected? and why?<br>Terminology such as 'deprivation decile' (line 258) needs explanation, especially for an international audience. Also, line 261 - 'lists of codes' needs explanation.	We have changed "routine clinical data" to "routinely collected healthcare records data" and added an explanation of what this data is, changed "Lists of codes" to "Lists of clinical codes (recorded as part of GP consultations)", and changed "deprivation decile" to "index of multiple deprivation decile" and added reference.
1.7	Line 289 - regarding the qualitative sampling strategy and use of purposive sampling - were you aiming to look for/ascertain differences between practices and between types of staff role in the ways the template was implemented? Could you explain	By sampling based on the 'different ways practices implemented the template' we mean that we sampled on the ways that practices fitted the combined reviews into their existing processes (for example, some practices did the review in two parts,

	further e.g. what were the different ways of implementing the template - and how did you know how this was being done?	others in one; some had experience with combined reviews already, some did not.) – we ensured we had interview participants from practices that followed these different processes. We knew how practices were implementing the reviews because this was part of the initial study set up discussion and subsequent check-in meetings with researchers.<br><br>Similarly with staff roles, we ensured that we interviewed staff from a range of administrative and clinical roles so that we could capture a wider picture of the impacts of implementing the template.<br><br>We have changed the relevant sentence to say<br>"Staff at implementation practices were purposively sampled to capture different ways practices implemented the template, and different staff roles."
1.8	Qualitative interviews: How were participants selected and approached? Over what time period did the interviews take place? I think each participant was interviewed once, but the section starting at line 504 seems to refer to staff views changing over time, please could you clarify?<br>The sentence starting in line 298 seems incomplete.	We have added two sentences to the section 'Qualitative data collection and analysis' stating that:<br><br>"Potential participants were sent an invite by the research lead at the practice, on behalf of the study team. Interviews took place between October 2022 and October 2023."<br><br>Later on in the paper we report staff views changing over time. This is based on staff members' own reflections of their changing opinions/experiences during the study period. It is correct that participants were only interviewed once.

1.9	Discussion:<br>Line 524 - sentence beginning 'Patients with multimorbidity' - I didn't understand this sentence.	Thankyou, we have edited the sentence for clarity and it now reads: 'Almost 1 in 20 of the adult population in recruited practices had multimorbidity'
1.10	Line 545 - why would the practices recruited first have more motivation that control practices?	Thankyou for pointing this out. We agree that this is not very clear, and the point we are trying to make is made in the final sentence. We have removed the unclear sentence.
	Reviewer: 2<br>Prof. Zoe Kelson, University of Exeter	
2.1	"Primary care (GP practices) in three English regions" [Abstract]<br>and<br>"Practices in deprived areas were prioritised, because multimorbidity is more prevalent in these areas.(5) Template implementation occurred in 2022-3.(18) "<br>and<br>"No formal sample size calculation was made. Intervention and control practices were recruited with the intention of obtaining wide variation across practices in practice size, urban/rural location, and deprivation and ethnicity of patient populations"<br>and<br>"To compare implementation and control practices, we included patients who were registered at the practice at all three time points (1.1.21, 249 1.4.22, 30.6.23). "<br>Can the authors please comment on whether they consider the included patients and practices to be representative?	As a mixed methods study, we were more interested in variation between practices and how they responded to the intervention than in seeking to obtain a fully representative sample. We succeeded in recruiting practices with a range of characteristics and patient populations.<br>We only included patients registered at all three time points to facilitate the interpretation of the statistical analysis, but we recognise that by excluding patients who joined or left the practices (including those who had died) this reduces the representativeness of the patient population. Inclusions and exclusions are shown in Figure 2.
2.2	Can the authors please consider applying sample weights to adjust for the selection process of practices?	We chose to adjust models for demographic factors as fixed effects and also fit practice as a random effect, in order to adjust for the selection process of practices, rather than applying sample weights.

		We appreciate there are arguments both ways for this, but many papers have found they are often equivalent (e.g. Analytic Strategies to Adjust Confounding Using Exposure Propensity Scores and Disease Risk Scores: Nonsteroidal Antiinflammatory Drugs (NSAID) and Short-term Mortality in the Elderly - PMC) and as is our preference we chose model adjustment rather than sample weighting.
2.3	"Routine medical record data; NoMAD questionnaires and qualitative interviews in implementation practices"<br>and<br>"Age, sex, deprivation decile, ethnicity, and number and type of long-term conditions were extracted at patient level for all eligible patients"<br>Can the authors please confirm if there was any missing data?	There was no missing data in age, sex, or number/type of long-term conditions. However, 7/10,423 (<0.1%) of patients were missing IMD and 644/10,423 (6.2%) were missing ethnicity. Details of the population and missing data can be found in Additional File 6.<br><br>We have added a sentence describing patient characteristics and their missing data in the patient characteristics paragraph of the results:<br>"In short, 20.4% patients were aged 18-59 year and 79.6% were 60+ years, 55.3% were female, 88.1% were of white ethnicity, and 52.9% were in IMD quintiles 1-2 (least deprived); we obtained complete age and sex data for all patients; However, <0.1% (7 participants) were missing IMD and 6.2% were missing ethnicity."
2.4	" Quantitative data collection included the Normalisation MeAsure Development (NoMAD) questionnaire,(21) a 23-item survey for assessing implementation of complex interventions in healthcare from the perspective of professionals directly involved"<br>Can the authors please comment on whether they may be potential for self-	The NOMAD questionnaire is a measure of an individual staff member's perceptions. As such, the concept of self-reporting bias is of limited applicability, since there is no objective measure against which the results can be judged.

	reporting bias?	
2.5	"Normalisation Process Theory (NPT) seeks to understand how innovations become normalised in 174 healthcare settings"<br>and<br>"For qualitative analysis, we used May et al.'s NPT coding manual which combines NPT with the Context Mechanism-Outcome (CMO) configuration of realist evaluation studies…<br>In line with the NPT coding manual, our initial logic model (Figure 1) described the contextual factors we considered when developing the implementation strategy and intervention, the mechanisms by which both were anticipated to act, and the outcomes by which each would be evaluated. "<br>The qualitative approach is clearly described.	Thank you very much for this acknowledgment.
2.6	"Data analysis is summarised below and described in detail in Additional File 3. "<br>and<br>"Additional File 3: Detailed description of statistical analysis"<br>Can more detail please be moved into the main article?	It is inevitably a challenge to write a paper which describes a mixed methods study within a reasonable word count, and which provides enough detail to satisfy those who are interested in the qualitative methods and others who are more interested in the quantitative methods. We think we have provided sufficient detail in the paper about the quantitative measures to inform the general reader and have provided further details in the additional files for the benefit of those who would like more information.
2.7	"assessed in the implementation period in implementation sites at the patient level using multilevel logistic regression with practice as a random effect, and adjusted for demographics (age, sex, ethnicity and IMD) as fixed effects. Further models added each long-term condition in turn, to assess	Thank you very much for this acknowledgement.

	differences in template use by condition. "<br>and<br>"Using multilevel logistic regression we assessed whether use of personalised care sections of the template differed between pre and implementation periods, adjusting for control practices, and between intervention and control practices, in a difference-in-difference framework. Models were adjusted for demographics (as above) as fixed effects, and patient ID nested with practice ID as random effects. Fidelity was further modelled at the practice level as the percentage of patients at each practice with the outcome. "<br>Appropriate modelling methods have been applied.	
2.8	"Quantitative and qualitative results were considered side-by-side; the extent to which the data converged /diverged was discussed with analytic insights developed through discussion. "<br>Can the authors please consider performing a Delphi process to reach a consensus?	We do not think it would be appropriate to conduct a Delphi process amongst the staff in our research team who were involved in this synthesis because this consensus method is not a recognised method of data integration in mixed methods studies. The approach we have used of developing analytic insights iteratively through discussion, informed by joint displays is standard practice of integration of qualitative and quantitative findings in mixed-methods research.<br>We have added the following reference to the paper providing rationale for this approach.<br>Achieving integration in mixed methods designs-principles and practices - PubMed
2.9	"Fifty-six staff from 13 practices completed the NoMAD questionnaire. Agreement with statements about coherence was generally high, particularly among nurses and healthcare assistants (Additional File	Thank you, we agree that this is a limitation, and have added a sentence in the discussion to say:<br><br>We did not conduct formal sample size calculations in this study, and

	7)"<br>Whilst no formal sample size calculation was conducted, can the authors please discuss sample size in the study limitations?	acknowledge that our sample of fifty-six staff completing the NoMAD questionnaire may impact the reliability of the survey results.
2.10	"The patients in whom the template was used were broadly similar to the cohort of all eligible patients (Additional File 6)."<br>and<br>"It was less well used in younger patients, those in least deprived areas and in eligible patients with epilepsy, mental health problems, dementia or learning difficulties, but more likely to be used in patients with CVD or with diabetes (Additional File 9)."<br>and<br>"There was a clear trend of greater familiarity with greater use of the template"<br>Can statistical outcomes please be reported in support of study findings throughout the Results text (n.b. the above excerpts are for example only)?	We have deliberately tried to include detailed statistical outcomes sparingly within the main paper. To include details of comparison groups, confidence intervals and p values for each of the points made in these examples would make the paper considerably longer and less readable. Instead, we have summarised the findings and referred the reader to tables in the additional files if they would like the full information.
2.11	"Because this is a non-randomised study, there could be differences between implementation and control practices which impact on our conclusions" [Strengths and Limitations of this study]<br>Can the Discussion of the study limitations please be expanded on?	We have expanded the limitation as follows:<br><br>“Because this is a non-randomised study, there could be unmeasured differences between implementation and control practices, such as patient and/or staff characteristics, which we were unable to account for, and could have therefore impacted on our conclusions.”
